# The Gut–Skin Axis in Pediatric Atopic Dermatitis: Nutritional Modulation, Microbiome Interactions, and Emerging Precision Approaches

**DOI:** 10.3390/nu18193138

**Published:** 2026-09-23

**Authors:** Raluca-Gabriela Miulescu, Mihnea Costescu, Ruxandra-Cristina Marin, Alina Mușetescu, Călin Muntean, Ioana Roşca, Iris-Iuliana Adam, Alina Ilici, Oana Andreia Coman

**Affiliations:** 1 Faculty of Medicine, University of Medicine and Pharmacy “Carol Davila”, 020021 Bucharest, Romania; miulescuraluca@yahoo.com (R.-G.M.); mihnea.costescu@umfcd.ro (M.C.); oana.coman@umfcd.ro (O.A.C.); 2 Saint Constantin Hospital, 500388 Brasov, Romania; adamirisiuliana@gmail.com; 3 Department of Dermatology, “Dr. Victor Babeş” Clinical Hospital of Infectious and Tropical Diseases, 030303 Bucharest, Romania; 4 Department of Dermatology, “Titu Maiorescu” University, 031593 Bucharest, Romania; 5 Department III Functional Science, Discipline of Medical Informatics and Biostatistics, “Victor Babes” University of Medicine and Pharmacy, 300041 Timisoara, Romania; cmuntean@umft.ro; 6 Pediatrics Department, National Institute for Mother and Child Health “Alessandrescu-Rusescu”, 20382 Bucharest, Romania; 7 Adeomed Clinic, 061344 Bucharest, Romania; driilici@yahoo.com

**Keywords:** pediatric atopic dermatitis, gut–skin axis, gut microbiome, nutrition, microbial metabolites, precision nutrition, probiotics, biomarkers

## Abstract

Background/Objectives: Pediatric atopic dermatitis (AD) is a barrier-driven inflammatory skin disease with systemic immune and microbial correlates. The gut–skin axis offers a biologically plausible framework through which early-life nutrition, gut microbial metabolism, intestinal barrier function, and immune maturation may influence disease risk and expression, but much of the supporting evidence remains associative. This narrative review asks which nutritional strategies are supported by clinical evidence in children with AD, which remain investigational, and how far the field is from precision nutrition. Methods: PubMed/MEDLINE, Scopus, and Web of Science were searched up to 31 July 2026; guidelines, systematic reviews, randomized trials, birth cohorts, and mechanistic studies with pediatric relevance were prioritized and appraised narratively. Results: Breastfeeding when possible, dietary diversity during complementary feeding, avoidance of unnecessary elimination diets, correction of documented nutritional deficiencies, and management of confirmed food allergy are supported by current guidance. Probiotics and synbiotics lower SCORAD in meta-analyses, but the pooled effect is small, heterogeneous, and below the minimal clinically important difference, so they are best described as an optional adjunct rather than a recommended treatment; Mediterranean-style or high-fiber dietary patterns are backed mainly by observational data. Microbiome profiling, metabolomics, multi-omics endotyping, and artificial intelligence remain research tools without validated predictive value for dietary responses. Conclusions: Nutrition is an adjunct to, not a substitute for, established topical and systemic therapy in pediatric AD. Precision nutrition is a promising research direction rather than an established component of routine care, and its clinical value will depend on standardized biomarkers and well-designed longitudinal and interventional studies.

## 1. Introduction

Pediatric atopic dermatitis (AD) is one of the most common chronic inflammatory disorders of childhood [1,2]. Epidermal barrier dysfunction and cutaneous type 2 inflammation remain central to its pathogenesis, and topical and systemic anti-inflammatory therapy remains the basis of treatment [3]. Over the past decade, however, systemic immune and microbial correlates of the disease have received growing attention, and the gut–skin axis has been proposed as a framework linking intestinal microbial ecology, nutrition, and immune development with cutaneous inflammation [1,4,5]. This framework is biologically plausible, yet it is still investigational: much of the evidence is associative, derived from small or heterogeneous studies, and not always reproducible. Nutrition is one of several modifiable exposures acting on this axis. It is best regarded as a candidate adjunct to established management rather than as a validated therapeutic pillar, and the distinction between experimental mechanism, observational association, and clinical effect demonstrated in trials is maintained throughout this review.

### 1.1. From Skin Disease to Systemic Inflammatory Disorder

The global prevalence of atopic dermatitis in children ranges from 10% to 20% in many high-income settings, with evidence of increasing burden in urbanizing populations worldwide [6]. Affected children and their families experience substantial impairment in quality of life, chronic sleep disruption, psychological morbidity, and significant healthcare utilization [7,8,9,10]. In the Romanian context, pediatric atopic dermatitis contributes meaningfully to the workload of primary care, dermatology, and allergology services, particularly in regions undergoing rapid dietary and environmental transitions [11,12]. In addition to this burden, the disease is clinically and biologically heterogeneous, which has motivated interest in more individualized approaches to its characterization and management [13,14].

### 1.2. Clinical Characteristics and Heterogeneity of Pediatric Atopic Dermatitis

Pediatric atopic dermatitis is characterized by remarkable clinical heterogeneity, with substantial variation in age at onset, lesion distribution, disease severity, persistence, and associated allergic comorbidities [13,15]. Approximately 60% of affected children develop symptoms during the first year of life, and nearly 85% present before the age of five years [13,16], underscoring the importance of early-life biological and environmental exposures in disease initiation. Although spontaneous remission occurs in a considerable proportion of patients during late childhood, many experience a chronic or relapsing course extending into adolescence and adulthood [17,18]. This variability has important clinical implications because children with apparently similar cutaneous manifestations at diagnosis may follow markedly different disease trajectories, reflecting differences in the underlying biological mechanisms that drive disease progression [18].

The clinical phenotype also evolves with age. In infancy, lesions involve the cheeks, scalp, and extensor surfaces as poorly demarcated, oozing, intensely pruritic plaques [13,19]; later they shift to the flexures, wrists, and neck, where scratching produces lichenification and xerosis [13,19]; adolescents often have localized but chronic disease of the face, hands, eyelids, or head and neck, with greater psychosocial burden and treatment resistance [16,17]. At every age, pruritus is the cardinal symptom, driving the itch–scratch cycle that perpetuates barrier disruption and inflammatory activation [15].

Beyond its cutaneous manifestations, pediatric atopic dermatitis imposes a multidimensional burden. Nocturnal pruritus causes sleep fragmentation, fatigue, and impaired cognition and behavior, and flares affect school attendance, social participation, and overall quality of life [9,20,21]. Parents report emotional stress and considerable costs associated with long-term treatment [8,20], and healthcare utilization adds indirect societal costs [6,8].

Increasing recognition of this clinical diversity has shifted attention from descriptive phenotypes toward biologically defined endotypes. Traditional phenotypic classifications based on disease severity, age of onset, or allergen sensitization remain clinically useful but provide limited insight into therapeutic responsiveness [14,22]. Contemporary research instead emphasizes the identification of endotypes characterized by distinct combinations of barrier dysfunction, immune dysregulation, microbial alterations, genetic susceptibility, and environmental influences [14]. Such stratification provides a conceptual basis for more individualized management. Whether it also explains the modest average effects of nutritional interventions in randomized trials is a hypothesis that has not been tested directly [23]. Consequently, understanding pediatric atopic dermatitis as a heterogeneous disorder rather than a single disease entity is a prerequisite for any future precision nutrition approach.

The age-dependent evolution of lesion distribution, clinical manifestations, and progression along the atopic march is summarized schematically in Figure 1 [13,24,25,26,27].

### 1.3. The Evolution of Pathophysiological Understanding

The marked clinical heterogeneity described above reflects equally complex biological mechanisms that have progressively reshaped the understanding of pediatric atopic dermatitis over recent decades. The conceptual framework for atopic dermatitis has evolved through successive layers of understanding. Early emphasis on the skin barrier highlighted the role of filaggrin mutations, reduced ceramides, and increased transepidermal water loss as primary initiators of inflammation [13]. Subsequent work established the central importance of type 2 immune dysregulation, with IL-4, IL-13, IL-31, and TSLP driving barrier impairment, IgE production, pruritus, and chronic inflammation [28]. Genetic susceptibility loci identified through genome-wide association studies further refined risk prediction but still left substantial unexplained variance [13,29]. A more recent development has been the incorporation of the gut microbiome and its metabolites into this model [1,4]. Several birth cohorts have reported differences in gut microbial composition, and in some cases in fecal metabolites, months before the clinical onset of AD [30,31,32]. Temporal precedence is compatible with a causal contribution but does not establish it, and no universally reproducible pediatric microbial signature has been identified; findings differ across cohorts, geographic settings, and sequencing methods [32,33]. Mechanistic work, largely in experimental models, has shown that microbial metabolites can influence keratinocyte differentiation, tight junction integrity, and regulatory T cell induction [34,35]. Nutrition is a major environmental exposure shaping the developing microbiome from pregnancy through the first two years of life [36,37], which makes it a plausible, and modifiable, point of intervention. Whether dietary modification actually alters the course of AD is a separate question. This is addressed in this review by distinguishing three levels of evidence: experimental mechanism, observational association, and clinical effect demonstrated in randomized trials or meta-analyses [19,23,38].

### 1.4. Nutrition as a Modifiable Environmental Determinant

Dietary exposures exert influence on immune and microbial development from the earliest stages of life. Maternal diet during pregnancy shapes the fetal immune environment and the initial microbial inoculum [36], human milk oligosaccharides promote *Bifidobacterium* species and tolerance-related metabolites [39,40], and the timing and diversity of complementary feeding, followed by dietary patterns throughout childhood, shape microbial succession and resilience [37,41]. Unlike genetic or fixed early-life factors, nutrition remains modifiable at every stage [42]. Recognizing nutrition as a regulator of microbiome maturation and immune development has motivated interest in moving from generalized dietary advice toward strategies adapted to the biological characteristics of each patient, although such strategies remain largely untested [14,43].

### 1.5. Toward Precision Nutrition and Precision Medicine

The logical response to heterogeneity is to match interventions to individual biology. In AD, treatment already moves from emollients and topical anti-inflammatory therapy toward phenotype-guided systemic treatment, and precision medicine aims to add molecular, microbial, and nutritional information to this process [22,43]. Precision nutrition, defined here as dietary intervention tailored to an individual’s microbial and metabolic profile, is the nutritional counterpart of this concept [43,44]. In pediatric AD it remains a research direction rather than an achieved clinical stage: no microbiome-guided dietary intervention has yet been validated in children. The present review is therefore framed around a critical question rather than a premise: how plausible, how well supported, and how close to clinical readiness is nutrition-based modulation of the gut–skin axis in pediatric AD? Its specific objectives are (i) to summarize critically the mechanisms of the gut–skin axis that have pediatric relevance; (ii) to rank nutritional interventions according to the strength of clinical evidence rather than biological plausibility; (iii) to delineate biomarkers, multi-omics, and computational approaches as candidate tools whose predictive value is unproven; and (iv) to propose a practical framework for nutritional assessment and counseling that can be applied with current evidence. Compared with recent reviews of the gut–skin axis or of dietary management in AD [1,2,3,4,5,45,46,47,48,49], this review adds an explicit evidence hierarchy applied consistently from mechanism to clinical recommendation, a pediatric clinical framework, and a statement of the limits of translation toward precision nutrition.

## 2. Materials and Methods

This narrative review was based on a structured literature search and a critical narrative synthesis addressing nutrition, the gut–skin axis, microbiome science, and precision approaches in pediatric AD. Electronic searches were performed in PubMed/MEDLINE, Scopus, and Web of Science Core Collection. The search covered publications from 1 January 2018 to 31 July 2026, with the last update performed on 31 July 2026; earlier landmark publications were retained when required to describe fundamental mechanisms or the origin of the gut–skin axis concept. No language restriction was applied at the search stage, but only articles published in English were included.

Searches combined Medical Subject Headings (MeSH) and free-text terms in the title and abstract fields using Boolean operators and truncation. The complete PubMed string was: (“dermatitis, atopic”[MeSH] OR “atopic dermatitis”[tiab] OR eczema[tiab]) AND (child*[tiab] OR pediatric[tiab] OR paediatric[tiab] OR infant*[tiab] OR “Child”[MeSH]) AND (“gastrointestinal microbiome”[MeSH] OR “gut microbio*”[tiab] OR “gut-skin axis”[tiab] OR “skin microbio*”[tiab] OR dysbiosis[tiab] OR probiotic*[tiab] OR prebiotic*[tiab] OR synbiotic*[tiab] OR postbiotic*[tiab] OR “short-chain fatty acid*”[tiab] OR indole*[tiab] OR “bile acid*”[tiab] OR metabolom*[tiab] OR “multi-omics”[tiab] OR “machine learning”[tiab] OR “artificial intelligence”[tiab] OR biomarker*[tiab]) AND (diet*[tiab] OR nutrition*[tiab] OR breastfeed*[tiab] OR “complementary feeding”[tiab] OR “Mediterranean diet”[tiab] OR “food allergy”[tiab] OR “elimination diet”[tiab] OR “vitamin D”[tiab] OR “omega-3”[tiab] OR “precision nutrition”[tiab] OR “personalized nutrition”[tiab]). Equivalent strings adapted to the syntax of Scopus (TITLE-ABS-KEY) and Web of Science (TS=) are provided in Supplementary Table S1. Reference lists of included reviews and guidelines were screened manually for additional publications.

Eligibility criteria were defined a priori. Included were international clinical practice guidelines and consensus statements, systematic reviews and meta-analyses, randomized controlled trials, prospective and birth-cohort studies, and mechanistic or translational studies conducted in children or with explicit pediatric relevance. Excluded were studies restricted to adults without a justified extrapolation to children, case reports and case series without mechanistic data, non-peer-reviewed preprints, conference abstracts, and primary studies superseded by more recent systematic syntheses. Titles and abstracts were screened independently by two authors (R.-G.M. and C.M.), full texts of potentially eligible records were assessed by the same two authors, and disagreements were resolved by discussion with a third author (O.A.C.). For each included publication the following information was extracted into a shared spreadsheet: population and age range, intervention or exposure, comparator, outcomes, study design, sample size, main findings, and limitations. Where several publications addressed the same question, recent systematic reviews, meta-analyses, and randomized trials were given precedence over smaller or older primary studies, in order to limit double counting of overlapping evidence.

Because this is a narrative review, no PRISMA flow diagram, formal risk-of-bias assessment, or quantitative synthesis was undertaken, and record counts at each screening stage were not formally tracked; the final reference list contains 159 publications. Methodological robustness was appraised narratively on the basis of study design, sample size, adequacy of control groups, consistency of findings across studies, biological plausibility, and relevance to pediatric practice. Throughout the manuscript, evidence is classified into three levels: experimental mechanism, observational association, and clinical effect demonstrated in randomized trials or meta-analyses. Descriptors such as ‘moderate’ or ‘low’ used in the tables reflect this narrative appraisal and do not correspond to formal GRADE certainty ratings. They are applied consistently across Table 1, Table 2, Table 3, Table 4 and Table 5 with the following meaning: ‘high’ denotes concordant conclusions from clinical practice guidelines, systematic reviews, or several randomized trials conducted in children; ‘moderate’ denotes randomized or prospective pediatric evidence that is limited in volume, statistically heterogeneous, or of uncertain clinical magnitude; ‘low’ denotes evidence restricted to observational studies, experimental models, or extrapolation from other populations. A descriptor qualifies the evidence for an effect and not the size of that effect, which is reported separately wherever it is known. The objective was to provide a clinically oriented synthesis, to identify evidence gaps, and to state where the field stands with respect to nutrition-guided precision approaches.

The evidence identified through this search is synthesized in the following sections, beginning with the biological mechanisms linking nutrition, microbial ecology, immune regulation, and epithelial barrier function within the gut–skin axis.

## 3. Biological Basis of the Gut–Skin Axis

Advances in microbiome science have added a systemic dimension to the understanding of pediatric AD, which nevertheless remains primarily a disease of the epidermal barrier and cutaneous immunity [3,84]. Within this broader view, the gut–skin axis describes how intestinal microbial communities and their metabolic activity may influence immune homeostasis, epithelial integrity, and cutaneous inflammation [85,86]. These mechanisms provide the biological rationale for nutrition-based interventions, although rationale alone does not establish clinical efficacy [87]. The following sections examine the principal components of the gut–skin axis: early-life microbiome development, intestinal barrier function, immune regulation, microbial metabolites, and gut–skin microbial crosstalk.

### 3.1. The Gut–Skin Axis: A Bidirectional Communication Network

The gut–skin axis describes the gastrointestinal tract and the skin as an interconnected network that communicates through immune mediators, microbial metabolites, endocrine signaling, and neural pathways, with consequences for immune tolerance, epithelial integrity, and inflammatory regulation [45,47,85]. The intestinal microbiome occupies a central position in this network. It matures rapidly during infancy under the influence of delivery mode, breastfeeding, complementary feeding, antibiotics, infections, and diet, and it shapes mucosal and systemic immunity through regulatory pathways, barrier maturation, and metabolite production; disruption of this process may impair tolerance and favor type 2 inflammation and allergic disease [71,81,87,88,89,90].

Communication along the axis relies on microbial metabolites, in particular short-chain fatty acids, indole derivatives, and secondary bile acids, which regulate T-cell differentiation, cytokine production, epithelial barrier function, and antimicrobial peptide expression, and on intestinal permeability, which may allow microbial products to reach the circulation and amplify cutaneous immune activation [43,46,81,90]. The relationship is not unidirectional: cutaneous inflammation may itself alter intestinal microbial composition through cytokine signaling, neuroendocrine stress pathways, dietary behavior, and treatment. The coexistence of intestinal dysbiosis and skin microbial alterations, particularly *Staphylococcus aureus* overgrowth, further supports the concept of an integrated host–microbiome ecosystem rather than isolated microbial compartments [49,91].

### 3.2. Early-Life Gut Microbiome Development and Immune Programming

Microbial colonization begins at birth and develops rapidly during infancy, in parallel with maturation of the intestinal barrier and the immune system. This period is often described as a ‘window of opportunity’ during which microbial exposures have lasting effects on immune tolerance and allergic susceptibility, and perturbations during it have been linked with later atopic dermatitis [92].

The composition of the infant gut microbiome is shaped by a complex interplay of host and environmental factors. Mode of delivery influences the initial microbial inoculum, with vaginally delivered infants acquiring microbial communities enriched in maternal vaginal and intestinal bacteria, whereas cesarean delivery is frequently associated with delayed colonization by beneficial anaerobic taxa and reduced microbial diversity during early life. Feeding practices represent another major determinant of microbial maturation. Human milk provides not only nutrients but also immunoglobulins, antimicrobial peptides, cytokines, and human milk oligosaccharides (HMOs). HMOs are not utilized by all bifidobacteria: they select for specific metabolic capabilities encoded at the species and strain level, such as those of *Bifidobacterium longum* subsp. *infantis*, so that the resulting community is typically of low taxonomic diversity but of a composition considered favorable for the breastfed infant [39,87]. The introduction of complementary foods, and in particular the cessation of breastfeeding, is followed by an increase in microbial diversity and functional complexity [32]. This increase is a normal developmental transition and should not be read as automatically beneficial; diversity is not in itself a marker of health at this age [28,32,87,92].

Antibiotics, infections, dietary transitions, household environment, pets, geography, and other lifestyle factors continue to shape microbial composition and function throughout childhood. Communities become more stable with age, but early perturbations may have persistent immunological consequences [81].

Several birth-cohort studies have reported that differences in early-life microbial composition precede the onset of AD, which argues against their being merely a consequence of established disease [30,31,37]. Infants who subsequently develop atopic dermatitis frequently exhibit reduced microbial diversity, lower abundance of short-chain fatty acid-producing bacteria, including *Faecalibacterium* and *Roseburia*, and delayed maturation of the gut microbial ecosystem. These microbial alterations have been associated with impaired immune tolerance, enhanced type 2 immune responses, and increased risk of allergic sensitization, and are compatible with a contributory role of intestinal dysbiosis in disease pathogenesis [37,39]. The picture is not uniform, however. In a longitudinal analysis of 1453 stool samples from 440 children, associations between fecal microbiota and later AD were present only at some time points and for some taxa after adjustment for confounders [32], and randomized prevention trials of *Lacticaseibacillus rhamnosus* GG in high-risk infants found no reduction in eczema incidence [93,94]. Such negative and inconsistent results indicate that the reported associations are context-dependent rather than a fixed pediatric signature.

The influence of the early-life microbiome extends beyond taxonomic composition alone. Increasing attention has shifted toward the functional capacity of microbial communities, including their ability to produce bioactive metabolites, regulate epithelial barrier integrity, and interact with the developing immune system. Consequently, contemporary research increasingly emphasizes microbial function rather than individual bacterial taxa as a determinant of health and disease. A caveat applies here: in most pediatric cohorts, microbial function has not been measured directly but inferred from 16S rRNA gene profiles using predictive tools such as PICRUSt2, which estimate gene content from taxonomy and cannot capture strain-level variation or actual gene expression [95]. Only studies that quantify metabolites, for example fecal short-chain fatty acids by targeted metabolomics [30,31], provide a measured rather than inferred readout of microbial activity. This functional perspective provides the biological rationale for nutritional strategies aimed at promoting beneficial microbial ecosystems during infancy and early childhood, although the mechanisms through which microbial metabolites regulate immune homeostasis are discussed in subsequent sections [37,39,90].

### 3.3. Intestinal Barrier Dysfunction and Immune Regulation

The intestinal epithelial barrier is a single cell layer joined by tight junctions, covered by mucus, and supported by immune cells, antimicrobial peptides, and resident microbiota. Together these components allow nutrient absorption while preventing microbial translocation and regulating the encounter between luminal antigens and the immune system [96,97].

Dysbiosis may impair tight junctions, reduce mucus production, and increase permeability, allowing lipopolysaccharide, peptidoglycan, and bacterial DNA to enter the circulation and sustain low-grade inflammation through innate immune receptors. The clinical significance of this ‘leaky gut’ in pediatric atopic dermatitis is still being investigated; current evidence suggests that barrier dysfunction may contribute to susceptibility and persistence in a subset of patients [34,35,80,98].

The intestinal barrier is closely integrated with the mucosal immune system, where continuous interactions between epithelial cells, dendritic cells, macrophages, innate lymphoid cells, and lymphocytes regulate the balance between immune activation and immune tolerance. In healthy individuals, microbial signals promote the differentiation of regulatory T (Treg) cells and the production of anti-inflammatory cytokines such as interleukin (IL)-10 and transforming growth factor-β (TGF-β), thereby maintaining immunological homeostasis. Intestinal dysbiosis may weaken these regulatory pathways and thereby favor an immune environment that is permissive to type 2 responses [80,99]. This should not be equated with a direct induction of the cytokine panel that characterizes AD. IL-4, IL-5, IL-13, IL-31, and thymic stromal lymphopoietin (TSLP) are produced predominantly in the skin by keratinocytes, skin-resident and skin-homing lymphocytes, and innate lymphoid cells [28], and no human study has demonstrated that gut dysbiosis directly induces this panel. The contribution of the gut is better described as a modulation of systemic tolerance and of the threshold for cutaneous type 2 activation [100,101]. These cytokines, whatever their site of origin, contribute to eosinophilic inflammation, elevated immunoglobulin E (IgE) production, impairment of epithelial barrier function, and persistent pruritus [28,102].

Importantly, intestinal and cutaneous barrier dysfunction are increasingly recognized as interconnected rather than independent processes. Type 2 cytokines, acting locally in the skin, impair epidermal barrier integrity by reducing filaggrin expression, altering ceramide synthesis, and suppressing antimicrobial peptide production. The resulting barrier disruption facilitates allergen penetration, microbial colonization, and recurrent inflammatory flares, creating a self-perpetuating cycle in which epithelial dysfunction and immune dysregulation reinforce one another. Consequently, intestinal barrier alterations may indirectly contribute to skin inflammation through immune-mediated mechanisms even in the absence of overt gastrointestinal disease, although the effects of type 2 cytokines on the skin do not by themselves indicate that these cytokines originated in the gut [30,102].

Current evidence is consistent with the concept that epithelial barrier dysfunction may extend beyond the skin in a subset of children with atopic dermatitis involving coordinated interactions between the intestinal epithelium, immune system, and resident microbiota. Understanding these mechanisms provides a biological framework for interventions aimed at restoring intestinal homeostasis through nutritional modulation of the gut microbiome. The following section examines the central role of microbial metabolites as the principal molecular mediators linking intestinal microbial activity with systemic immune regulation and skin health [30].

### 3.4. Microbial Metabolites as Central Mediators of the Gut–Skin Axis

The functional activity of the microbiome may matter more than its taxonomic composition. Metabolites generated by microbial fermentation act as signaling molecules on epithelial barrier integrity, immune cell differentiation, and inflammatory pathways, and they are the principal biochemical link between the gut and distant organs, including the skin [79,103].

Short-chain fatty acids (SCFAs), mainly acetate, propionate, and butyrate, are the best-characterized of these mediators. Produced by fermentation of dietary fiber, they act locally, where butyrate fuels colonocytes and reinforces tight junctions and mucus production, and systemically, where SCFAs promote regulatory T (Treg) cell differentiation, restrain pro-inflammatory pathways, and increase interleukin (IL)-10 production. In experimental models, SCFAs also enhance filaggrin expression, ceramide synthesis, and keratinocyte differentiation, which provides a mechanistic link between intestinal fermentation and skin barrier function [30,33,39].

Tryptophan-derived indole derivatives, including indole-3-lactic acid (ILA), indole-3-carboxaldehyde (3-IAld), and indoxyl sulfate, activate the aryl hydrocarbon receptor (AhR) in epithelial and immune cells, supporting barrier maintenance and antimicrobial peptide production while limiting type 2 inflammation. Altered tryptophan metabolism has been reported in children with atopic dermatitis, which makes this pathway a candidate contributor and therapeutic target [103].

Secondary bile acids, polyamines, and microbial lipids are further candidates. Secondary bile acids signal through the farnesoid X receptor (FXR) and Takeda G protein-coupled receptor 5 (TGR5) and thereby influence inflammation and epithelial homeostasis; their role in pediatric atopic dermatitis is incompletely understood, although metabolomic studies suggest that altered bile acid metabolism may contribute to disease heterogeneity [15,79].

The effects of these metabolites depend on dietary substrate availability as much as on microbial composition. Fiber, human milk oligosaccharides, polyphenols, and fermented foods support communities with the metabolic capacity to produce SCFAs and indoles, so that nutrition acts on the gut–skin axis mainly by changing microbial function. This helps explain heterogeneous responses to dietary interventions among children with similar microbial composition [18]. The principal diet–microbiome–metabolite pathways linking intestinal and skin barrier function, immune regulation, and the development of atopic dermatitis are summarized in Figure 2.

Recent advances in metabolomics have further transformed the understanding of microbiome–host interactions by enabling comprehensive characterization of the biochemical products generated by the intestinal microbiota. Metabolomics does not, however, identify microbial activity directly. Many metabolites detected in feces or plasma, including short-chain fatty acids, indoles, and bile acids, have combined dietary, host, and microbial origins, and their concentrations also reflect absorption, host metabolism, and medication [80]. Attributing a metabolite signal to the microbiota therefore requires complementary approaches such as stable-isotope tracing, integration with metagenomic data, or experimental validation. This approach is considered a candidate component of precision nutrition because it may yield functional biomarkers; whether such biomarkers can predict disease severity or dietary responses in children has not been established [49,104].

Collectively, these findings indicate that microbial metabolites are a plausible molecular interface through which nutrition influences immune regulation and skin homeostasis. Understanding the functional consequences of microbial metabolism therefore provides the mechanistic foundation for microbiome-targeted nutritional strategies and is one prerequisite for any future precision nutrition approach for pediatric atopic dermatitis [49,55,104].

## 4. Pediatric Atopic Dermatitis Beyond the Skin

Although pediatric atopic dermatitis is primarily a cutaneous disease, it is frequently accompanied by systemic immune, microbial, and psychosocial correlates. In a subset of children, it is also the earliest manifestation of a broader atopic predisposition involving epithelial barrier dysfunction, immune maturation, microbial homeostasis, and environmental interactions. These interconnected processes influence not only disease severity but also the subsequent development of allergic and non-allergic comorbidities throughout childhood. Consequently, understanding the natural history and systemic consequences of pediatric atopic dermatitis provides the clinical context for individualized nutritional and microbiome-directed management strategies discussed in the following sections [15,100].

### 4.1. The Natural History and the Atopic March

The clinical course of pediatric atopic dermatitis is heterogeneous. Many children improve or remit during late childhood, whereas others follow a persistent course with recurrent flares, increasing treatment requirements, and additional allergic disorders. Birth cohort studies suggest that these divergent outcomes are often set during early immune and microbial development, so that the disease is better regarded as a developmental disorder than as a static diagnosis [105,106].

The concept of the atopic march describes the observation that atopic dermatitis often precedes food allergy, allergic rhinitis, or asthma [100,101]. These conditions do not follow a fixed sequence; they are better described as components of several possible longitudinal patterns, in which any of them may occur first, alone, or in combination [18,100]. Nevertheless, children with early-onset, severe, and persistent disease consistently demonstrate a substantially greater risk of subsequent allergic comorbidities. Defective epidermal barrier function facilitates epicutaneous sensitization to environmental and food allergens, initiating type 2 immune responses that may subsequently extend to other epithelial organs. At the same time, early alterations in gut microbial development and immune tolerance may further increase susceptibility to allergic sensitization, reinforcing the concept that multiple epithelial barriers contribute to disease evolution [20,21].

The atopic march is therefore not a linear progression but a network of developmental pathways shaped by genetic susceptibility, microbial ecology, nutrition, environment, and immune maturation. Some children have isolated atopic dermatitis, whereas others develop asthma or food allergy without significant eczema; individualized risk assessment and early identification of children at risk of persistent disease follow from this diversity of trajectories [20,21,105,106,107].

Among the biological factors associated with these trajectories, the gut microbiome has attracted particular attention. Longitudinal studies have associated reduced microbial diversity, depletion of butyrate-producing bacteria, altered microbial metabolite production, and impaired intestinal barrier function during early life with both persistent atopic dermatitis and progression toward additional allergic disorders. As discussed in Section 3, these alterations may interfere with immune tolerance during critical developmental windows, providing a biologically plausible explanation for the lasting influence of early-life nutritional exposures on allergic disease susceptibility [20,21,106,107,108]. Two limitations should be stated plainly. Exploratory studies are highly variable, and no validated microbiome-based tool can currently identify which children will progress along any of these trajectories [33,109]. Moreover, no trial has demonstrated that a nutritional intervention modifies the natural history of atopic dermatitis [23,38].

Recognition of the developmental nature of pediatric atopic dermatitis has important implications for clinical practice. Rather than focusing exclusively on short-term control of cutaneous inflammation, contemporary management increasingly aims to identify children at greatest risk of persistent disease and progression along the atopic spectrum. This perspective supports early interventions that preserve epithelial barrier integrity, promote immune tolerance, and maintain microbial homeostasis during periods of heightened developmental plasticity. Nutrition is often proposed as a disease-modifying strategy on this basis, but this remains a hypothesis to be tested rather than an established effect, as discussed in the sections that follow [104].

### 4.2. Clinical and Molecular Heterogeneity

Pediatric atopic dermatitis is not a single disease entity but a spectrum of biologically distinct disorders sharing a clinical phenotype. Children with similar eczema distribution and severity differ in the relative contributions of barrier dysfunction, immune activation, microbial dysbiosis, genetic susceptibility, and environmental exposure, which explains much of the variability in onset, persistence, treatment response, and comorbidity and limits uniform management [80].

Four terms are used in this section and throughout the manuscript with distinct meanings. A clinical phenotype is a set of observable features such as age at onset, distribution, severity, and persistence. Allergic sensitization refers to the detection of allergen-specific IgE by skin prick test or serum assay; it is a laboratory finding that is neither a phenotype nor an endotype in itself and does not equal clinical allergy. A molecular endotype is a subgroup defined by a distinct pathobiological mechanism, for example a dominant type 2 or Th17/Th22 inflammatory program or a filaggrin-related barrier defect [14,22]. A microbial profile is a descriptive pattern of gut or skin microbial composition or activity; because such profiles have not been shown to define stable, mechanistically distinct subgroups, they are referred to here as candidate microbial profiles rather than as endotypes.

Traditionally, pediatric atopic dermatitis has been classified according to observable clinical characteristics, including age at onset, disease severity, anatomical distribution, and chronicity, with the presence or absence of IgE sensitization used as an additional stratifier (the so-called extrinsic and intrinsic forms). Early-onset disease developing during infancy is generally associated with greater severity, increased persistence, and a higher likelihood of progression along the atopic march than disease first appearing later in childhood. Likewise, children with extensive or persistent eczema frequently exhibit greater systemic immune activation and more pronounced barrier dysfunction than those with localized or intermittent disease. Although these clinical phenotypes remain valuable for diagnosis and prognostic assessment, they provide only limited insight into the biological mechanisms driving disease expression [13,39].

Endotypes classify patients by shared molecular and immunological pathways rather than clinical appearance. Type 2 inflammation, with increased IL-4, IL-13, IL-5, IL-31, thymic stromal lymphopoietin (TSLP), and serum IgE, remains dominant in most children, but Th17, Th22, and in some populations Th1 pathways contribute to heterogeneity, particularly in chronic, severe, or ethnically diverse groups, and influence epidermal differentiation, microbial colonization, and response to targeted therapy [49].

Barrier integrity is a second source of heterogeneity. Loss-of-function variants in the filaggrin (FLG) gene remain among the strongest genetic risk factors, yet many children with significant disease carry no FLG mutation, and others develop barrier dysfunction secondary to inflammation-induced suppression of structural proteins, illustrating the interaction between genetic susceptibility and immune-mediated barrier impairment [104].

Beyond host genetics and immune dysregulation, increasing attention has focused on candidate microbial profiles. Longitudinal studies have demonstrated substantial interindividual variation in both gut and skin microbial composition, with some children exhibiting depletion of short-chain fatty acid-producing bacteria, whereas others are characterized predominantly by *Staphylococcus aureus* overgrowth or alterations in microbial metabolic activity. Some of these microbial differences may precede clinically apparent disease [31,55], which is compatible with a role in early immune programming, although reverse causation and confounding by feeding, antibiotics, and environment cannot be excluded [32]. Consequently, children with apparently similar clinical severity may differ considerably in their underlying microbial ecology and biological mechanisms of disease [49,55].

The principal clinical phenotypes, sensitization-based subgroups, molecular endotypes, and candidate microbial profiles of pediatric atopic dermatitis, together with their predominant biological characteristics and potential implications for nutrition-guided management, are summarized in Table 1.

### 4.3. Beyond the Skin: Systemic Comorbidities

Recognition of pediatric atopic dermatitis as a systemic inflammatory disorder has expanded clinical attention beyond cutaneous manifestations to encompass the broad spectrum of comorbidities that frequently accompany the disease. Although chronic eczema and pruritus remain its defining clinical features, affected children commonly experience allergic, infectious, gastrointestinal, psychological, and sleep-related complications that substantially contribute to disease burden and long-term health outcomes. These comorbidities are heterogeneous in origin [55,105,106]. Allergic comorbidities share immunological mechanisms with atopic dermatitis, whereas sleep disturbance and psychological morbidity are largely consequences of pruritus, chronic disease burden, and visible lesions; the latter are clinically important but do not by themselves demonstrate generalized immune dysregulation [7,20,21].

Food allergy, allergic rhinitis, and asthma are the most extensively studied comorbidities, and children with early-onset, severe, or persistent atopic dermatitis are at increased risk of developing one or more of them. Epicutaneous sensitization through a defective barrier and altered immune tolerance both contribute, but progression is highly variable and differs considerably among individual patients [20,21,107].

Sleep disturbance represents another major yet frequently underrecognized consequence of pediatric atopic dermatitis. Persistent nocturnal pruritus disrupts normal sleep architecture, resulting in reduced sleep duration, impaired sleep quality, daytime fatigue, irritability, and diminished cognitive performance. These effects frequently extend beyond the affected child, with caregivers experiencing repeated nocturnal awakenings, emotional stress, and reduced quality of life. Moreover, chronic sleep disruption has been proposed to amplify inflammatory responses through neuroimmune mechanisms [110], establishing a self-perpetuating cycle in which worsening inflammation aggravates pruritus and further compromises sleep [105].

Children with moderate-to-severe disease have higher rates of anxiety, depressive symptoms, impaired self-esteem, and social withdrawal, and visible lesions, itch, and demanding treatment interfere with school and peer relationships. Parents experience emotional distress, caregiver burden, and financial strain. Family-centered care should therefore address psychological and social well-being alongside skin treatment [7,10,20].

Functional gastrointestinal disorders, altered intestinal permeability, and intestinal dysbiosis are reported more often in affected children than in controls. Causality is not established, but these observations are consistent with the bidirectional gut–skin axis discussed in Section 3 [104].

These manifestations call for multidisciplinary care involving dermatologists, allergists, pediatricians, dietitians, psychologists, and primary care physicians, extending beyond control of skin inflammation to barrier care, treatment of allergic comorbidities, psychosocial support, and nutritional assessment. Within this framework, nutrition is one adjunctive component of comprehensive, patient-centered management [49,104].

The principal systemic manifestations and multidimensional consequences of pediatric atopic dermatitis are summarized in Figure 3, illustrating both immune-mediated comorbidities and the consequences of chronic disease burden.

## 5. Nutrition Shapes the Gut–Skin Axis

Building upon the biological mechanisms discussed in the previous sections, this section examines how nutritional exposures influence gut microbial ecology, microbial metabolism, immune regulation, and clinical outcomes throughout childhood. Particular emphasis is placed on nutritional strategies capable of modifying the gut–skin axis and their potential integration into precision nutrition approaches for pediatric atopic dermatitis.

### 5.1. From Precision Medicine to Precision Nutrition

Conventional treatment decisions rely on clinical severity, lesion distribution, and age. These remain essential, but they only partly capture the biological mechanisms of disease: children with similar phenotypes differ in genetic background, immune endotype, barrier function, microbiome, nutritional status, and exposures, and their disease course and treatment response differ accordingly. These observations explain the interest in precision medicine for pediatric atopic dermatitis, although its clinical realization is still limited [22,111].

Precision medicine seeks to tailor therapeutic strategies according to the biological characteristics of individual patients rather than relying exclusively on observable clinical features. In pediatric atopic dermatitis, this multidimensional approach integrates clinical phenotype with molecular endotypes, immune signatures, genetic susceptibility, microbiome composition, and environmental influences to better define the mechanisms driving disease in each child. The introduction of targeted therapies, including monoclonal antibodies directed against IL-4 and IL-13 signaling and Janus kinase (JAK) inhibitors, has substantially improved outcomes in moderate-to-severe disease [111,112]. Their success should not, however, be read as evidence of individualized treatment selection based on a validated endotype. In current practice these agents are chosen according to age, disease severity, response to previous treatment, comorbidities, accessibility, and safety, not according to a molecular or microbial biomarker [111,113]. Endotype-guided selection therefore remains a goal rather than an achievement, for pharmacological and nutritional therapy alike.

The transition toward precision nutrition is increasingly supported by advances in microbiome sequencing, metabolomics, transcriptomics, proteomics, and computational biology. These technologies may in time help to identify endotypes and biomarkers relevant to nutritional interventions. At present, baseline microbial composition, microbial metabolite profiles, vitamin D status, intestinal permeability, dietary quality, and inflammatory markers should be regarded as candidate predictors or exploratory biomarkers: they have been associated with disease or with treatment outcomes in individual studies, but none has been validated as a predictor of response to a nutritional intervention in children [23,52,76].

### 5.2. Early-Life Nutritional Programming

Nutritional exposures from conception through the first two years of life, the so-called first 1000 days, shape the establishment of microbial communities and their metabolic activity, epithelial barrier maturation, and immune tolerance. Because this window is biologically plastic, early nutrition is particularly relevant to long-term allergic outcomes [62,114].

Among the earliest nutritional influences, breastfeeding remains one of the most consistently supported protective factors against pediatric atopic dermatitis. Beyond providing optimal nutrition, human milk contains immunoglobulins, antimicrobial peptides, cytokines, and human milk oligosaccharides (HMOs), which selectively promote the growth of *Bifidobacterium* species and support the development of a gut ecosystem considered favorable for the breastfed infant. In the exclusively breastfed infant this ecosystem is typically dominated by a few *Bifidobacterium* species and is of low taxonomic diversity; greater diversity in this period is not in itself a sign of better health and may accompany earlier cessation of breastfeeding [32,87]. Through the generation of acetate, lactate, and subsequent cross-feeding of butyrate-producing bacteria, these microbial communities contribute to immune tolerance, epithelial barrier maturation, and regulation of inflammatory responses. Although the magnitude of protection against atopic dermatitis varies among studies and observational data are prone to confounding [63], exclusive breastfeeding during early infancy is recommended on the basis of its overall benefits, with a possible, though inconsistently demonstrated, protective effect against atopic dermatitis [63,83,115].

The transition to complementary feeding represents a second critical stage of nutritional programming. The gradual introduction of diverse plant-derived foods, legumes, fruits, vegetables, whole grains, and fermented products expands the availability of fermentable substrates, which is accompanied by an increase in microbial diversity and in the production of fermentation-derived metabolites. Conversely, limited dietary diversity during infancy has been associated in observational studies with an increased risk of allergic sensitization and allergic disease [67]; the causal pathway, and the extent to which it runs through the microbiome, remain to be established. Contemporary infant feeding recommendations therefore emphasize timely introduction of a wide variety of complementary foods while avoiding unnecessary dietary restrictions in children without confirmed food allergy [83].

Early nutrition acts alongside maternal diet in pregnancy, delivery mode, antibiotic exposure, and feeding practices. These factors cannot always be modified, but nutrition remains among the most accessible of them, and it influences the gut microbiome as an ecological system rather than through isolated nutrients [36,87,114].

Collectively, these observations indicate that early-life nutrition influences pediatric atopic dermatitis not only through direct nutritional effects but also by shaping the developing microbiome and its metabolic capacity. This ecological perspective provides the foundation for understanding how broader dietary patterns, rather than individual nutrients alone, regulate the gut–skin axis throughout childhood [116,117].

### 5.3. Gut Dysbiosis in Pediatric Atopic Dermatitis

Accumulating evidence indicates that children with atopic dermatitis exhibit recurrent, though not fully consistent, alterations in gut microbial composition compared with healthy peers, which are compatible with a contributory role of intestinal dysbiosis in disease pathogenesis. Longitudinal birth-cohort studies have demonstrated that these microbial alterations may be detectable during the first months of life, preceding the onset of clinically apparent eczema and being associated with disease development and persistence. These observations suggest that the gut microbiome participates in early immune programming and may influence long-term allergic trajectories [118].

The microbial signature most consistently associated with pediatric atopic dermatitis includes reduced bacterial diversity, depletion of major short-chain fatty acid (SCFA)-producing taxa such as *Faecalibacterium prausnitzii* and *Roseburia* species, and relative enrichment of potentially pro-inflammatory microorganisms, including selected members of the *Enterobacteriaceae*, *Clostridium*, and *Streptococcus* genera. These alterations are generally more pronounced in children with early-onset, moderate-to-severe, or persistent disease and in those presenting with food sensitization or other allergic comorbidities. Several caveats limit the interpretation of these findings. Most derive from 16S rRNA gene sequencing of fecal samples, which yields relative rather than absolute abundances, so that a reported enrichment or depletion of a taxon does not establish an absolute increase or decrease [33,95]. Fecal samples do not necessarily represent the mucosa-associated microbiota that interacts most directly with the immune system. The results are further influenced by age, delivery mode, feeding, antibiotic exposure, geography, and sequencing and bioinformatic methods, which contributes to the inconsistency across cohorts [32,119]. The observation that microbial differences can precede clinical disease is compatible with a causal contribution but does not prove it.

Microbial function may be more informative than composition: children with similar taxonomic profiles can differ substantially in their capacity to produce SCFAs, indoles, and secondary bile acids, the metabolites that regulate barrier integrity and immune tolerance (Section 3). Disruption of microbial metabolism may therefore explain disease heterogeneity more directly than changes in bacterial abundance [31].

The skin microbiome shows parallel changes, with reduced diversity and *Staphylococcus aureus* overgrowth closely associated with severity and flares. Gut and skin communities are distinct ecosystems but are connected through systemic immune signaling and circulating metabolites, so dysbiosis is better viewed as a multisite disturbance of host–microbiome homeostasis [91].

For nutritional research, the implication is that interventions should be judged by their ability to restore microbial function and metabolite production, not simply to increase diversity; this perspective frames the evaluation of dietary patterns, probiotics, synbiotics, and vitamin D in the next section [120].

### 5.4. Nutritional Factors Regulating the Microbiome and Clinical Outcomes

Among the various strategies proposed to modulate the gut–skin axis, nutritional interventions currently represent the most accessible and clinically applicable approach. However, the strength of evidence differs considerably according to the intervention evaluated, with randomized evidence, of modest effect size, available for probiotics and synbiotics and for vitamin D supplementation in deficient children, and mainly observational evidence for plant-rich dietary patterns [23]. Collectively, these interventions seek to restore microbial homeostasis, reinforce epithelial barrier function, and attenuate immune dysregulation, although clinical benefits remain variable among individual patients [23].

The 2024 EAACI Task Force systematic review and meta-analysis is the most comprehensive synthesis of randomized evidence on nutrition-based interventions in children with atopic dermatitis without confirmed food allergy. It included 27 randomized placebo-controlled trials, and in a meta-analysis of 20 of them probiotics alone or combined with prebiotics produced a statistically significant reduction in SCORAD; the evidence on prebiotics alone, vitamin D, evening primrose oil, and partially hydrolyzed whey formula was judged too limited to support a recommendation [23]. An umbrella synthesis of 38 earlier meta-analyses is compatible with these findings: pre-, pro-, and synbiotics were associated with a lower incidence of atopic dermatitis (relative risk 0.74, 95% CI 0.70–0.79) and with a mean SCORAD reduction of 3.75 points (95% CI 2.42–5.08). That reduction is well below the minimal clinically important difference of 8.7 SCORAD points, so the pooled estimate should not be presented as a clinically meaningful improvement for the average child [59,121]. In subgroup analyses of the same synthesis, multi-strain probiotics, synbiotics, and mixed preparations lowered SCORAD whereas prebiotics alone did not, and the reduction was confined to moderate-to-severe disease; these comparisons are exploratory, were not designed as tests of effect modification, and do not identify responders [59].

These findings emphasize an important principle emerging across contemporary research: the success of nutritional interventions depends not only on the intervention itself but also on the biological characteristics of the individual child. Baseline microbiome composition, microbial metabolic capacity, nutritional status, disease endotype, and environmental exposures are increasingly recognized as factors contributing to the substantial variability observed among intervention studies. It is often argued that the modest average effects reported in meta-analyses conceal clinically meaningful responses in biologically distinct subgroups [64]. This remains a hypothesis: observed variability between individuals does not by itself demonstrate heterogeneity of the treatment effect, and no trial has yet identified a subgroup of children in whom a nutritional intervention is reliably effective [23].

Beyond supplements, overall dietary quality matters. Mediterranean-style patterns rich in vegetables, fruits, legumes, whole grains, olive oil, nuts, and fermented foods are associated with greater microbial diversity and metabolite production, whereas Western patterns rich in ultra-processed foods, refined carbohydrates, saturated fats, and added sugars are associated with a less diverse, more pro-inflammatory microbiome. Such patterns act on the microbiome as an ecological system rather than through single nutrients [56].

Individual components contribute through complementary mechanisms: fiber supplies fermentable substrate, polyphenols have prebiotic-like effects, and omega-3 fatty acids influence membrane lipids and pro-resolving mediators, although clinical evidence for omega-3 supplementation is less consistent than for dietary patterns as a whole. Sustained improvement in dietary quality is therefore likely to have more durable effects than isolated supplementation [38,56,65,122].

Current evidence therefore supports a transition from isolated nutritional interventions toward integrated dietary approaches that consider both microbial ecology and host biology. To summarize the principal nutrition–microbiome interactions relevant to pediatric atopic dermatitis, Table 2 summarizes the reported nutrition–microbiome associations for the major nutritional factors, their primary microbial targets, proposed mechanisms, associated clinical outcomes, the predominant type of supporting evidence, and research priorities [38]. Because this review is narrative and did not apply formal risk-of-bias assessment or certainty grading, the table is a structured summary rather than an evidence map.

Although growing evidence supports the role of nutrition in modulating the gut–skin axis, dietary management in pediatric atopic dermatitis frequently extends beyond supplementation and dietary patterns. Questions surrounding food allergy, elimination diets, oral tolerance, and dietary diversity remain among the most common challenges encountered in clinical practice and are discussed in the following section [23,38,56,59,64,65,122].

### 5.5. Food Allergy and Dietary Management in Pediatric Atopic Dermatitis

Food allergy and atopic dermatitis often coexist, particularly in infants with early-onset, moderate-to-severe disease, but the relationship is more complex than once believed. Barrier impairment facilitates epicutaneous sensitization and IgE-mediated food allergy, yet eczema alone is not evidence of clinically relevant food allergy, and sensitization on skin prick testing or serum-specific IgE frequently occurs without symptomatic reactions [50].

Four situations are often conflated and should be distinguished, because they call for different dietary decisions [68,70,123]. Allergic sensitization is the presence of allergen-specific IgE without a proven clinical reaction, and it does not justify avoidance on its own. IgE-mediated food allergy is a reproducible, usually immediate reaction to a food, confirmed by history, testing, and, when necessary, oral food challenge; here avoidance of the culprit food is indicated. Non-IgE-mediated food allergy comprises cell-mediated disorders such as food protein-induced enterocolitis syndrome and allergic proctocolitis, which are diagnosed clinically and by elimination and reintroduction rather than by IgE testing [124]. Finally, food-triggered eczema, in which a food reproducibly worsens atopic dermatitis in the absence of an immediate reaction, is uncommon, is over-diagnosed on the basis of parental report, and should be confirmed by supervised elimination and reintroduction before any long-term restriction is advised [70,123].

International guidelines discourage empirical elimination diets in children without convincing evidence of food allergy. Unnecessary restriction risks nutritional deficiency, impaired growth, reduced dietary diversity, and psychosocial burden, and, from the perspective of the gut–skin axis, it removes fermentable substrates needed for microbial diversity and metabolite production, potentially compromising rather than improving immune tolerance [102].

Conversely, current evidence supports the timely introduction of common allergenic foods such as peanut and well-cooked egg during infancy, in line with international allergy guidelines. Early introduction promotes oral tolerance and adds dietary diversity; this shift from avoidance to immune education is one of the most important advances in pediatric allergy prevention of the past decade [51].

When food allergy is confirmed by history, testing, and, when indicated, supervised oral food challenge, elimination remains essential. Restriction should be individualized, reassessed periodically, and accompanied by dietetic counseling to secure adequate energy, protein, calcium, vitamin D, and iron, since prolonged elimination in young children may affect growth, nutritional status, and microbiome maturation [66].

Taken together, current evidence supports a selective rather than restrictive approach to dietary management in pediatric atopic dermatitis. Nutritional interventions should aim to preserve dietary diversity, maintain adequate nutritional status, support microbial homeostasis, and promote immune tolerance whenever possible, while reserving elimination diets for children with clearly established food allergy. This selective strategy is guided by clinical and allergological evidence; it does not depend on microbiome or biomarker data and can be applied with the tools available today [51,66,102].

### 5.6. The Skin Microbiome and Its Interaction with Gut Signals

The skin microbiome is an equally relevant component of the gut–skin axis. Intestinal and cutaneous communities communicate through systemic immune signaling, circulating metabolites, endocrine pathways, and host inflammation, so interventions that modify the gut microbiome may also affect the microbial ecology of the skin indirectly [49].

Healthy skin is colonized by commensals such as *Staphylococcus epidermidis*, *Cutibacterium* spp., and *Corynebacterium* spp., which support barrier integrity and antimicrobial defense. In atopic dermatitis this community loses diversity and is overgrown by *Staphylococcus aureus*, whose proteases, exotoxins, and superantigens impair the barrier and amplify type 2 inflammation, in close association with severity and flares [2].

Although direct nutritional modulation of the skin microbiome remains an emerging field, accumulating evidence suggests that improvements in gut microbial function may indirectly promote cutaneous microbial homeostasis. As discussed in Section 3, microbial metabolites, including short-chain fatty acids, indole derivatives, and secondary bile acids, modulate systemic immune responses and epithelial barrier integrity, thereby, at least in experimental systems, creating an environment that is less favorable for *S. aureus* overgrowth and chronic cutaneous inflammation. This concept suggests that restoration of intestinal microbial function may influence skin health even without directly altering the composition of the cutaneous microbiota [125].

The reciprocal relationship between the gut and skin further reinforces this concept. Chronic cutaneous inflammation may influence intestinal permeability, dietary behaviors, and systemic inflammatory pathways, whereas intestinal dysbiosis may enhance skin inflammation through immune-mediated mechanisms. Rather than representing isolated pathological processes, alterations within the gut and skin microbiomes should therefore be viewed as interconnected manifestations of a broader disturbance in host–microbiome homeostasis [49,91].

Future microbiome-directed therapies are therefore likely to target the communication between microbial ecosystems, epithelial barriers, and the immune system rather than a single community, with nutrition acting as one contributor to systemic microbial resilience. The next section weighs the evidence for this approach [104,126,127].

### 5.7. Strength of Evidence, Current Limitations, and Future Directions

The cumulative evidence reviewed throughout this section supports the concept that nutrition is a modifiable exposure with a plausible, though incompletely demonstrated, influence on pediatric atopic dermatitis, acting through interconnected effects on gut microbial ecology, microbial metabolism, epithelial barrier integrity, and immune regulation. Mechanistic studies, birth-cohort investigations, observational research, randomized clinical trials, and recent meta-analyses provide biologically coherent, but largely associative, evidence that nutritional exposures are linked with disease susceptibility and clinical expression. Nevertheless, despite this rapidly expanding body of literature, the translation of these findings into standardized clinical recommendations remains limited by important methodological and biological challenges [23,38].

A major limitation of the current evidence is the substantial heterogeneity among published studies. Differences in patient age, disease severity, microbiome composition, baseline nutritional status, dietary assessment methods, intervention protocols, probiotic strains, supplementation doses, treatment duration, and outcome measures complicate direct comparisons across studies and contribute to inconsistent findings. These challenges are particularly evident in trials evaluating probiotics, synbiotics, and vitamin D supplementation, where modest average clinical benefits are accompanied by considerable interindividual variability in treatment response. Negative or inconclusive trials may reflect the heterogeneity of the study populations [57,128], but this explanation is itself unproven and should not be used to discount null results [93,94].

Most intervention studies have tested isolated nutrients or supplements rather than dietary patterns, although nutritional exposures rarely act independently within the gut ecosystem. Future trials should evaluate whole-diet approaches such as Mediterranean-style or high-fiber diets, with objective measures of adherence, microbiome composition, metabolites, immune markers, and clinical outcomes [72].

The field is also moving from descriptive microbiome research toward mechanism-based studies that use standardized sequencing, functional metagenomics, metabolomics, and longitudinal sampling to define endotypes, and toward validation of biomarkers that could identify children likely to benefit from specific interventions [109,129].

To facilitate translation of the current evidence into routine clinical practice, Table 3 summarizes practical nutrition-based recommendations according to common clinical scenarios encountered in pediatric atopic dermatitis, together with their underlying microbiome rationale and the current level of supporting evidence.

## 6. From Gut Dysbiosis to Precision Nutrition

The growing understanding of the gut–skin axis has broadened the conceptual framework of pediatric atopic dermatitis, but translating it into practice requires reliable methods to identify children likely to benefit from a given intervention and to monitor their response. Such a transition would depend on biomarkers, multi-omics technologies, and computational approaches; the following sections examine these tools and their potential role in the future implementation of precision nutrition [109,129].

### 6.1. Clinical and Molecular Biomarkers for Precision Nutrition

Precision nutrition depends on biomarkers that can identify biologically distinct subgroups, predict responsiveness to a nutritional intervention, and monitor outcomes. Children with similar clinical manifestations differ in immune activation, barrier integrity, microbial ecology, metabolism, and nutritional status, and biomarkers are the interface between these differences and individualized management [14,52,75].

Biomarkers serve different purposes, and the categories are not interchangeable. Following the BEST (Biomarkers, EndpointS, and other Tools) framework of the FDA-NIH Biomarker Working Group [73], a diagnostic biomarker detects or confirms disease; a prognostic biomarker identifies the likelihood of a clinical event or of progression irrespective of treatment; a predictive biomarker identifies individuals more likely to respond to a specific intervention; a monitoring biomarker is measured serially to assess disease status; a response (pharmacodynamic) biomarker shows that a biological response has occurred after an intervention; a susceptibility or risk biomarker indicates the potential for developing disease in currently unaffected individuals; and an exposure biomarker reflects contact with a dietary or environmental factor. Precision nutrition requires predictive biomarkers above all, and it is in this category that the evidence in pediatric atopic dermatitis is weakest. Most markers discussed below have been evaluated only as diagnostic, prognostic, or monitoring markers of the disease itself, not as predictors of dietary response. Table 4 assigns each marker to its BEST category.

No single biomarker captures this complexity, and multidimensional panels are increasingly favored. Clinical assessment remains the basis of stratification: SCORAD, EASI, and POEM provide standardized measures of severity and response, and age at onset, persistence, flare frequency, and comorbidities add prognostic information, although these measures give only indirect insight into the biological mechanisms of individual variability [11,14,130].

Circulating immune markers, including total IgE, eosinophil count, thymus and activation-regulated chemokine (TARC/CCL17), periostin, IL-13, IL-31, and TSLP, are associated with type 2 activity and severity. None is specific enough to guide nutritional interventions on its own; these markers contribute to immune endotyping; their use as predictive biomarkers of response to nutritional strategies is hypothetical [77,78].

Barrier-related markers evaluate the target organ directly: transepidermal water loss (TEWL), epidermal lipids, filaggrin expression, ceramide content, antimicrobial peptides, and minimally invasive tape stripping inform on epidermal integrity and local inflammation, and are most informative when combined with systemic markers [53].

Among the emerging biomarker domains, gut-derived biomarkers appear especially promising for nutrition-guided management. Shotgun metagenomic sequencing characterizes microbial diversity and the genetic potential of the community, whereas metabolomics captures the metabolites present in a sample, whatever their origin, including short-chain fatty acids, indole derivatives, and secondary bile acids. As discussed in Section 3, measured metabolites provide a more direct, though not microbe-specific, readout than taxonomy alone. Likewise, markers of intestinal permeability, including zonulin, lipopolysaccharide-binding protein (LBP), intestinal fatty acid-binding protein (I-FABP), and regenerating islet-derived protein 3 alpha (Reg3A), are candidate markers of gut barrier dysfunction; whether children identified by these markers benefit from targeted nutritional interventions is untested [131].

Nutritional markers complete the panel. Serum 25-hydroxyvitamin D [25(OH)D], the omega-3 index, habitual fiber intake, and measures of dietary quality describe modifiable factors that may influence microbial function and immune homeostasis, and they allow supplementation to be targeted rather than empirical [80,130].

Collectively, these complementary biomarker domains should not be regarded as competing alternatives but as interconnected components of a unified precision nutrition framework. Future clinical tools may integrate clinical phenotype, immune profiling, barrier assessment, microbiome composition, metabolomic signatures, and nutritional evaluation into predictive models, but such models do not yet exist for nutritional decisions in pediatric atopic dermatitis. Table 4 summarizes the principal candidate biomarker domains discussed in relation to precision nutrition in pediatric atopic dermatitis, highlighting representative biomarkers, their potential clinical utility, the current strength of evidence, and their readiness for implementation in clinical practice [79,85].

### 6.2. Multi-Omics Approaches to Endotyping Pediatric Atopic Dermatitis

Because no single biomarker captures the interactions among genetics, environment, nutrition, microbial ecology, immunity, and epithelial function, attention has turned to multi-omics, an integrative systems biology approach that combines complementary molecular datasets to characterize disease endotypes [132].

Genomics identifies inherited susceptibility, of which loss-of-function variants in the filaggrin (FLG) gene are the most extensively studied. Genetic predisposition explains only part of disease risk and little of the variability in phenotype, progression, or treatment response, so genetic data must be integrated with downstream molecular processes that reflect gene-environment interaction [54].

Transcriptomics and proteomics describe the biological activity behind disease expression. Gene expression profiling shows that inflammatory pathways differ among children and change over the disease course, and proteomics quantifies structural proteins, cytokines, chemokines, and antimicrobial peptides directly, giving a more complete picture of disease activity than genomic or clinical data alone [133].

Among the various omics technologies, metabolomics has emerged as one of the most promising for nutrition-guided medicine. Unlike microbial taxonomy, which describes community composition, and metagenomics, which describes genetic potential rather than gene expression or actual metabolic flux [95], metabolomics measures the metabolites present in a sample. Fecal or plasma metabolomes integrate contributions from diet, medication, the host, and the microbiota, so that a metabolite signature is not in itself a measure of microbial activity [80]. As discussed in Section 3, microbial metabolites, including short-chain fatty acids, indole derivatives, and secondary bile acids, represent the principal molecular mediators linking diet, the gut microbiome, and immune regulation. Metabolomic profiling may therefore complement, rather than replace, taxonomic and metagenomic data; whether it predicts responsiveness to dietary interventions in children has not been tested [54,132].

Parallel advances in microbiome science have further expanded this integrative framework. High-throughput sequencing now enables characterization of both intestinal and cutaneous microbial communities at unprecedented resolution, extending beyond taxonomic profiling to include microbial gene content and inferred functional pathways, which represent potential rather than realized activity [95]. Increasing evidence indicates that integrating microbiome sequencing with metabolomics provides a more complete representation of host–microbe interactions than either technology individually, thereby improving biological characterization of pediatric atopic dermatitis [134].

The principal strength of multi-omics lies not within any individual technology but in the integration of multiple biological layers into unified models capable of defining clinically meaningful disease endotypes. Combining genomic susceptibility, transcriptomic activity, proteomic signatures, metabolomic profiles, microbiome composition, nutritional assessment, and clinical phenotype provides a systems-level understanding of disease heterogeneity that cannot be achieved using isolated biomarkers. Such characterization is often described as the foundation of precision nutrition. It should be noted, however, that no multi-omics-derived subgroup has yet been shown to identify children who benefit from a specific dietary intervention; the studies available are cross-sectional or descriptive and have not been linked to interventional outcomes [132,133,134,135].

Despite these remarkable advances, several important challenges remain before multi-omics can be routinely incorporated into pediatric clinical practice. Standardization of laboratory methodologies, harmonization of bioinformatic pipelines, validation across ethnically diverse populations, demonstration of cost-effectiveness, and development of clinically interpretable analytical frameworks are all essential prerequisites for widespread implementation. Whether integrated multi-omics profiling will become a component of nutritional care in pediatric atopic dermatitis will depend on prospective studies that link omics-defined subgroups to differential responses to dietary interventions [136].

Figure 4 summarizes the systems biology framework underlying precision nutrition in pediatric atopic dermatitis, illustrating how environmental exposures, multi-omics technologies, biological pathways, and clinical endotypes converge to guide individualized nutritional interventions.

### 6.3. Current State of Precision Nutrition

Whether nutritional strategies can be directed toward children with identifiable biological characteristics is the central question of precision nutrition. The nearest approximation is vitamin D. A meta-analysis of eleven randomized trials in children and adults reported a reduction in disease severity with supplementation (standardized mean difference −0.41, 95% CI −0.67 to −0.16; I2 = 58%), whereas the EAACI task force considered the pediatric evidence too limited to support a recommendation [23,58]. Baseline vitamin D status was reported inconsistently and no trial randomized children according to serum 25-hydroxyvitamin D concentration, so the frequent claim that benefit is confined to deficient children remains plausible but untested; what is established is that documented deficiency should be corrected for reasons independent of the skin. By contrast, the proposal that microbiome-directed interventions are particularly effective in children with reduced microbial diversity or impaired microbial metabolic activity has no supporting evidence: observed variability in individual responses does not demonstrate true heterogeneity of the treatment effect, and no trial has stratified children by microbial features and shown a differential response to probiotics, prebiotics, or dietary fiber [23,59]. Dietary counseling can already take habitual dietary quality and fiber intake into account, but this is conventional nutritional practice rather than precision nutrition [38,67].

Several barriers limit implementation: predictive biomarkers are not validated, standardized microbiome and metabolomic analyses are not widely available, intervention studies use heterogeneous methods that hinder comparison and external validation, and evidence on long-term disease modification and prevention of allergic progression is scarce. Prospective longitudinal studies with standardized biological characterization are needed [113].

### 6.4. Artificial Intelligence and Machine Learning for Personalized Nutrition

Microbiome sequencing, multi-omics, metabolomics, and digital health generate data volumes that exceed conventional statistical analysis, and artificial intelligence (AI) and machine learning (ML) are proposed as tools to integrate them. In pediatric atopic dermatitis, AI is viewed as an enabling technology for stratification and prediction rather than as a therapy in itself [135].

Machine learning models have been applied to atopic dermatitis to classify disease from transcriptomic and microbial data, to predict short-term severity, and to explore endotypes [134,137]; no published model predicts responsiveness to a nutritional intervention in children. By simultaneously analyzing clinical phenotype, immune biomarkers, microbiome composition, metabolomic profiles, dietary intake, and environmental exposures, these models can identify complex biological patterns that are not readily detectable using conventional analytical methods. Identifying patterns in such data does not justify recommending specific diets [138]. Before any model could inform dietary decisions it would need external validation in independent cohorts, assessment of calibration and not only discrimination, safeguards against data leakage between training and test sets, decision-curve analysis to show net clinical benefit, and reporting according to TRIPOD+AI [139].

AI may also improve nutritional assessment and monitoring. Digital food records, smartphone applications, and wearable devices allow more objective, longitudinal assessment of intake and lifestyle, and their integration with biomarkers and clinical outcomes could support dietary advice that adapts to changes in disease activity and response over time [26,138,140].

Another promising application involves predictive modeling during early life. By integrating genetic susceptibility, nutritional exposures, microbiome development, immune biomarkers, and environmental factors, AI-based models may eventually identify infants at increased risk of persistent atopic dermatitis or progression along the atopic march. Such predictive approaches could facilitate earlier implementation of preventive nutritional strategies during periods of maximal immune and microbial plasticity, although no such model has been validated prospectively [140].

Robust models require large, diverse, well-characterized datasets, and differences in sequencing methods, dietary assessment tools, and outcome measures limit external validation. Data privacy, algorithm transparency, explainability, and regulatory oversight are further requirements. Any future AI system should function as a transparent decision-support tool that complements, rather than replaces, clinical judgment [141].

### 6.5. Challenges and Future Directions for Clinical Translation

Most evidence for microbiome-directed nutritional interventions comes from small studies with heterogeneous populations, diverse protocols, and short follow-up. Standardization of study design, sequencing and metabolomic methods, dietary assessment, and outcome measures is a prerequisite for reproducible evidence and meaningful comparison [109].

Few candidate biomarkers have undergone prospective validation sufficient for clinical use. Research should prioritize standardized biomarker panels that combine complementary biological domains while remaining practical, reproducible, and interpretable [142].

Biological complexity must be balanced against feasibility and equity. Comprehensive multi-omics profiling requires laboratory infrastructure, bioinformatic expertise, and funding that are concentrated in specialized centers, which creates a risk of unequal access. Implementation should therefore favor simplified panels of validated clinical markers and practical decision-support tools that can be applied across healthcare settings, supported by cost-effectiveness and implementation studies [143,144,145].

Translation also depends on collaboration among pediatricians, dermatologists, allergists, gastroenterologists, dietitians, microbiologists, and data scientists, and on the active participation of families, whose dietary preferences, cultural practices, and treatment goals should remain central to nutritional planning. Sustained adherence to healthy dietary patterns, rather than short-term supplementation, is likely to remain the basis of any microbiome-oriented management [146,147,148].

## 7. Translating Precision Nutrition into Clinical Practice

Advances in microbiome science have created opportunities to improve nutritional management, but implementation requires more than biological insight. Nutritional interventions must be integrated into dermatological care, adapted to each child’s clinical characteristics, nutritional status, and risk profile, and delivered through multidisciplinary collaboration. The following sections summarize the principles supported by current evidence and outline an author-proposed framework for nutritional assessment and counseling, while acknowledging that precision nutrition strategies remain under investigation [2].

### 7.1. Principles of Nutritional Management

Nutritional management is one component of comprehensive care, not a substitute for dermatological therapy. Dietary management may complement pharmacological treatment by supporting barrier integrity, nutritional adequacy, and overall health, and it should be individualized according to severity, nutritional status, age, growth, feeding practices, gastrointestinal symptoms, and confirmed food allergy [23].

Its goals extend beyond the skin: normal growth and development, nutritional adequacy, dietary diversity, and support of oral tolerance. Infants and young children with moderate-to-severe disease, gastrointestinal symptoms, prolonged restriction, feeding difficulties, or confirmed food allergy are at greatest risk of deficiency and impaired growth and deserve particular attention [69].

International recommendations support a diverse, balanced diet whenever medically appropriate: exclusive breastfeeding in early infancy, timely introduction of complementary foods, and progressive diversification. Mediterranean-style patterns rich in fruits, vegetables, legumes, whole grains, fermented foods, and fiber are consistent with the biology of the gut–skin axis and with general health, whereas empirical elimination diets should be avoided in children without confirmed food allergy [38].

Nutritional supplementation should likewise be evidence-based and individualized rather than routinely prescribed. Correction of documented nutritional deficiencies is justified in its own right because it protects growth, skeletal health, and nutritional status. For vitamin D specifically, repletion of children with documented deficiency is warranted on these grounds alone. Randomized evidence indicates that supplementation can reduce disease severity, but the trials are small and short, baseline vitamin D status is reported inconsistently, and vitamin D should not be presented to families as a treatment for atopic dermatitis in children who are vitamin D replete [23,58]. Probiotic and synbiotic preparations are not recommended as treatment for atopic dermatitis in current dermatological guidelines. Where families raise the question, a multi-strain probiotic or a synbiotic evaluated in randomized trials may be discussed as an optional adjunct in moderate-to-severe disease, provided that the expected average benefit is described as small, that no component of standard therapy is replaced, and that the preparation is stopped if no benefit is apparent after a predefined period [23,59,149,150]. Importantly, clinicians should communicate realistic expectations regarding the magnitude of benefit currently demonstrated for nutritional interventions and emphasize that these strategies are intended to complement, not replace, standard dermatological management [12,60,151]. In children requiring systemic therapy or prolonged dietary restriction, baseline and follow-up assessment should include growth parameters, the laboratory monitoring specified for the systemic agent used, and markers of nutritional adequacy whenever the diet is restricted [69,150].

The principal nutrition-based interventions currently investigated in pediatric atopic dermatitis differ in their proposed biological mechanisms, strength of evidence, and readiness for routine clinical implementation. Table 5 summarizes the principal nutrition-based interventions currently investigated in pediatric atopic dermatitis, highlighting their proposed mechanisms within the gut–skin axis, current evidence, practical clinical recommendations, and key priorities for future research [151].

### 7.2. Practical Nutritional Counseling for Children and Families

Parents often seek dietary advice at diagnosis and are exposed to conflicting information from social media and non-evidence-based sources. Nutritional counseling should therefore be part of routine care, providing individualized recommendations and correcting misconceptions that lead to unnecessary restriction or inappropriate supplementation [12].

Counseling starts with a structured assessment of growth, habitual intake, feeding practices, gastrointestinal symptoms, previous elimination diets, supplements, and confirmed food allergy. Children with moderate-to-severe disease, feeding difficulties, selective eating, multiple exclusions, or impaired growth are at increased nutritional risk, and early involvement of a pediatric dietitian improves adequacy and adherence while reducing both deficiency and unnecessary avoidance [123,124].

Counseling should emphasize dietary quality rather than restriction: age-appropriate diversity and sustainable patterns rich in fruits, vegetables, legumes, whole grains, and fermented foods, rather than ‘superfoods’ or commercial supplements. Supplementation should be considered only when clinically indicated or supported by evidence [51,70].

Addressing misconceptions regarding food allergy is equally important. Families should understand that worsening eczema does not necessarily indicate food allergy and that positive allergy tests alone do not establish clinical reactivity. Elimination diets should therefore be reserved for children with confirmed food allergy following appropriate clinical evaluation. Shared decision-making, supported by collaboration among pediatricians, dermatologists, allergists, and dietitians, remains essential for balancing nutritional adequacy with safe allergen avoidance while minimizing unnecessary dietary restrictions [51,70,123].

Counseling is a continuing process. Dietary habits, requirements, severity, and family circumstances change throughout childhood, and recommendations should be reassessed at follow-up and adapted to the child’s needs [70,124,149].

### 7.3. Integration with Pharmacological and Biologic Therapies

Nutritional interventions complement, and do not replace, pharmacological therapy. Emollients, topical anti-inflammatory treatment, and timely management of flares remain the basis of care, and biologic agents and Janus kinase (JAK) inhibitors have substantially improved control of moderate-to-severe disease. Dupilumab, which blocks interleukin-4 and interleukin-13 signaling, is approved for moderate-to-severe atopic dermatitis from 6 months of age, and selected oral Janus kinase inhibitors are approved for adolescents [111,112]. Nutritional strategies belong alongside these treatments within a multidisciplinary plan, not as alternatives to them [152].

Pharmacological and nutritional interventions address different clinical needs. Targeted therapies suppress dysregulated inflammatory pathways, whereas nutritional management aims to maintain growth, nutritional adequacy, and dietary diversity, and possibly to support barrier function and microbial resilience [150]. It is reasonable to combine them within one management plan, but the evidence for an additive or synergistic effect of nutrition on the outcome of pharmacological treatment is insufficient, and no trial has tested this combination directly [38].

Integration into routine care requires collaboration among pediatricians, dermatologists, allergists, gastroenterologists, and pediatric dietitians, so that dietary advice is adapted to severity, food allergy status, growth, gastrointestinal symptoms, deficiencies, and treatment response and remains aligned with the overall therapeutic plan [42].

### 7.4. A Proposed Framework for Nutritional Assessment and Counseling

Although comprehensive microbiome profiling and multi-omics technologies remain confined to research, several principles supported by current guidelines can already be incorporated into routine pediatric care. Management begins with a structured assessment of severity, growth, nutritional status, dietary diversity, gastrointestinal symptoms, and confirmed food allergy, which identifies children who need specialist referral or more detailed nutritional evaluation [67].

Subsequent nutritional management should prioritize the maintenance of a nutritionally adequate and diverse dietary pattern, correction of documented nutritional deficiencies, and avoidance of unnecessary elimination diets in children without confirmed food allergy. Adjunctive probiotic or synbiotic therapy with preparations evaluated in randomized trials may be discussed for selected children with moderate-to-severe disease, within comprehensive multidisciplinary management and as an option rather than a guideline-recommended treatment. Criteria such as gastrointestinal symptoms or recent antibiotic exposure are pragmatic prompts for such a discussion, not validated predictors of response, and ‘microbial imbalance’ has no standardized clinical definition and should not be used as a selection criterion. Regular follow-up should include monitoring of disease severity, growth, nutritional status, quality of life, and adherence to dietary recommendations [76,123].

Figure 5 summarizes an author-proposed framework for nutritional assessment and counseling in pediatric atopic dermatitis. It does not constitute precision nutrition, and it has not been validated prospectively. The framework emphasizes sequential clinical assessment, individualized nutritional decision-making, multidisciplinary collaboration, and longitudinal follow-up while acknowledging the future incorporation of microbiome biomarkers, metabolomics, and precision nutrition as these approaches become validated for routine clinical use.

Author-proposed framework for integrating nutritional assessment and counseling into pediatric atopic dermatitis management. Nutritional strategies are individualized according to clinical phenotype, food allergy status, nutritional assessment, and disease severity, microbiome biomarkers and precision nutrition approaches are shown as future, currently unvalidated, extensions.

The proposed framework reflects current guideline-based practice and the authors’ clinical experience; it should be viewed as a flexible aid rather than a prescriptive or validated protocol. As microbiome science, biomarker validation, and precision nutrition continue to evolve, future algorithms are expected to incorporate increasingly sophisticated biological information capable of supporting truly individualized nutritional care. Until such approaches become widely available, careful clinical assessment, evidence-based nutritional counseling, and multidisciplinary collaboration remain the cornerstones of nutrition-guided management in pediatric atopic dermatitis [76].

## 8. From Research to Clinical Practice: Future Outlook

### 8.1. Remaining Knowledge Gaps

Most intervention trials have measured changes in severity over weeks to a few months, and little is known about long-term disease modification, prevention of persistence, or interruption of the atopic march. Long-term randomized trials and prospective cohorts are needed to determine whether nutritional interventions produce sustained immunological and clinical benefit [119].

An additional research priority is the development of standardized core outcome sets for nutritional intervention studies in pediatric atopic dermatitis. Greater harmonization of clinical endpoints, dietary assessment methods, microbiome analyses, biomarker panels, and patient-reported outcomes would improve comparability across studies and strengthen the quality of future evidence [74].

### 8.2. Emerging Therapeutic Opportunities

Next-generation probiotics, defined microbial consortia, postbiotics, microbial metabolites, engineered strains, and personalized synbiotics are being investigated as more targeted alternatives to conventional probiotics, whose colonization and efficacy are variable. Their aim is a predictable functional effect on microbial metabolism and immune regulation; all remain investigational [153,154,155,156].

Nutritional metabolomics, continuous dietary monitoring, and digital health may in future allow dietary advice to adapt to changes in the microbiome, disease activity, and developmental stage rather than remain static [43,61,157].

## 9. Conclusions

Pediatric atopic dermatitis is primarily a barrier-driven inflammatory skin disease with systemic immune and microbial correlates. The gut–skin axis offers a biologically plausible framework linking dietary exposures, microbial metabolism, immune regulation, and cutaneous inflammation, but the supporting evidence is heterogeneous and largely associative, and nutrition is one of several modifiable exposures acting on this axis rather than its dominant determinant.

Current evidence supports the integration of nutrition into comprehensive disease management while emphasizing that dietary interventions should complement, rather than replace, established pharmacological therapies. Maintenance of dietary diversity, breastfeeding when possible, correction of documented nutritional deficiencies, and avoidance of unnecessary elimination diets are supported by current guidance. Mediterranean-style dietary patterns rest on observational data and on general child-health considerations rather than on trials in atopic dermatitis, and probiotic or synbiotic preparations rest on randomized evidence whose pooled effect is small, heterogeneous, and below the minimal clinically important difference; they remain an optional adjunct and not a recommended treatment. However, broader clinical implementation of precision nutrition will require standardized biomarkers, high-quality longitudinal clinical trials, and validation of microbiome-informed therapeutic strategies.

Microbiome science, multi-omics technologies, artificial intelligence, and nutritional research may in time make nutrition-guided management more individualized. For now, precision nutrition in pediatric atopic dermatitis is a research direction rather than an established component of care. Its future value will depend on standardized biomarkers, validated predictive tools, and well-designed longitudinal and interventional studies; whether it can contribute to preventing the progression of allergic disease is an open question rather than an expected outcome [23,38,134].

## Figures and Tables

**Figure 1:**
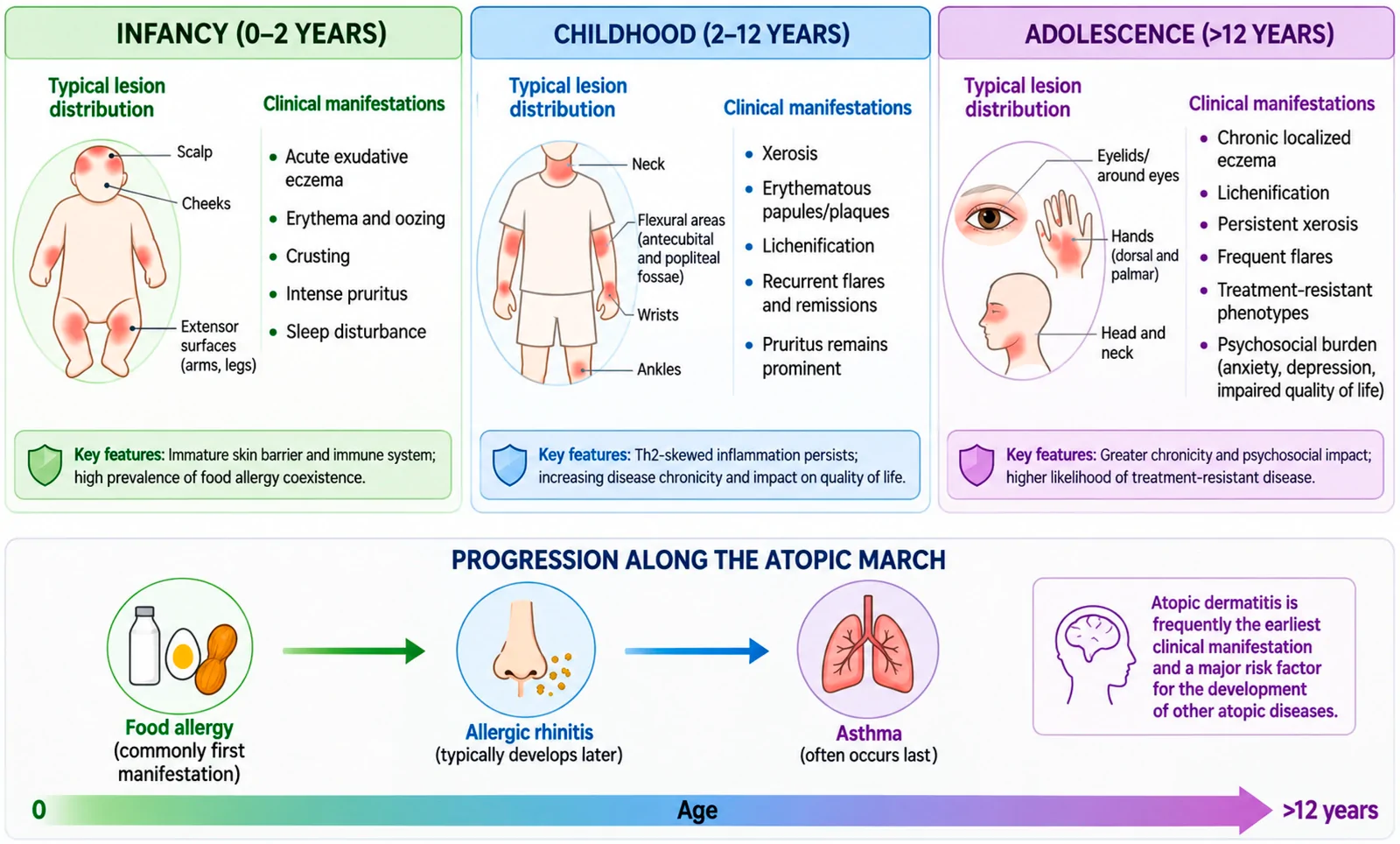
Age-dependent clinical evolution of pediatric atopic dermatitis and progression along the atopic march. Schematic overview of the age-dependent changes in lesion distribution and clinical manifestations of pediatric atopic dermatitis, illustrating the typical progression of the atopic march. Created using Inkscape (v0.92 and v1.0; Inkscape Project, https://inkscape.org, accessed on 10 September 2026).

**Figure 2:**
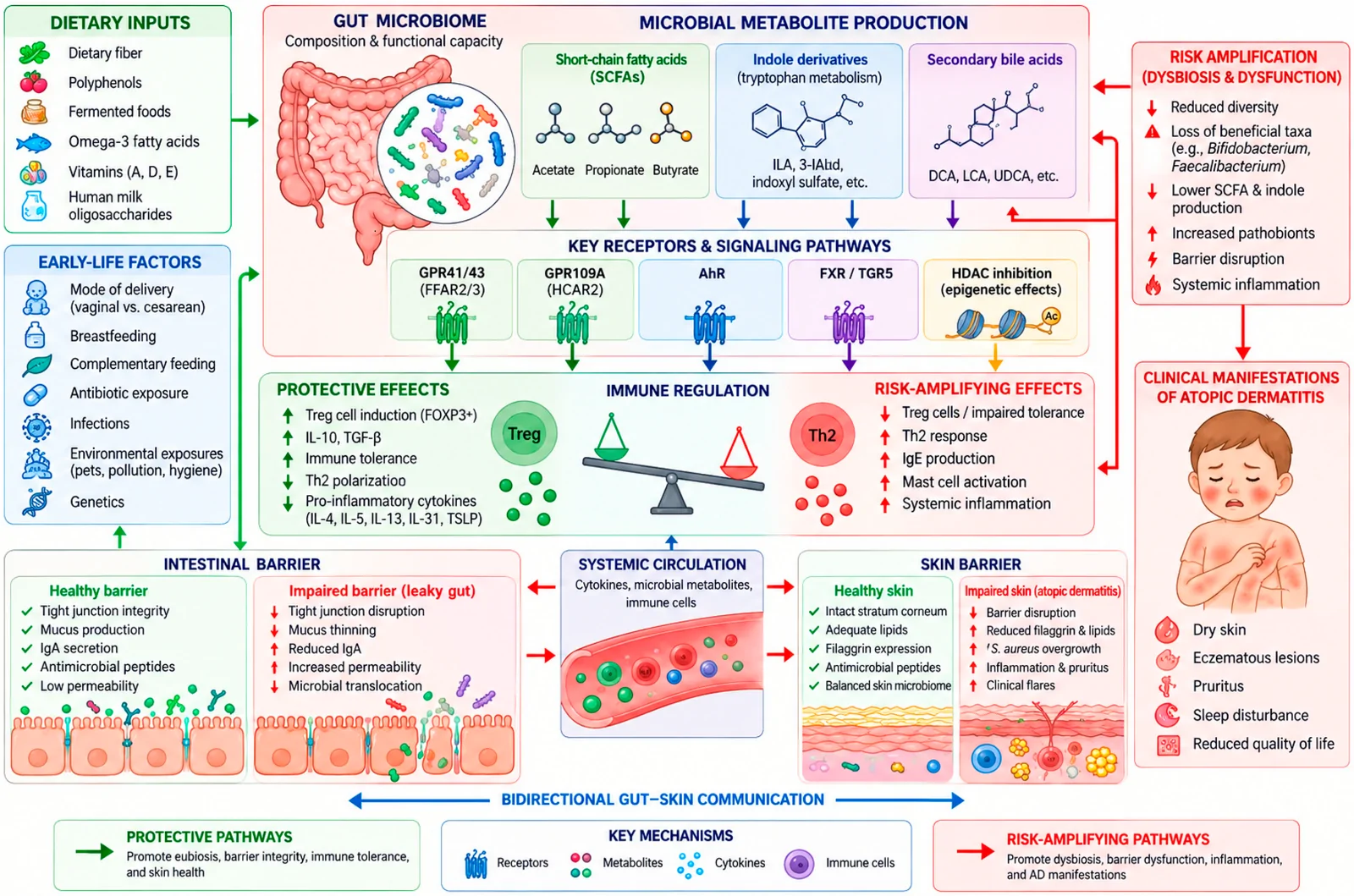
Mechanistic overview of the gut–skin axis in pediatric atopic dermatitis. Early-life factors and dietary exposures influence gut microbial composition and metabolite production, thereby regulating immune homeostasis, epithelial barrier integrity, and skin inflammation. Microbial metabolites, including short-chain fatty acids, indole derivatives, and secondary bile acids, act through multiple signaling pathways to modulate gut–skin communication, whereas dysbiosis promotes barrier dysfunction, type 2 inflammation, and the clinical manifestations of pediatric atopic dermatitis. Created using Inkscape (v0.92 and v1.0; Inkscape Project, https://inkscape.org, accessed on 10 September 2026).

**Figure 3:**
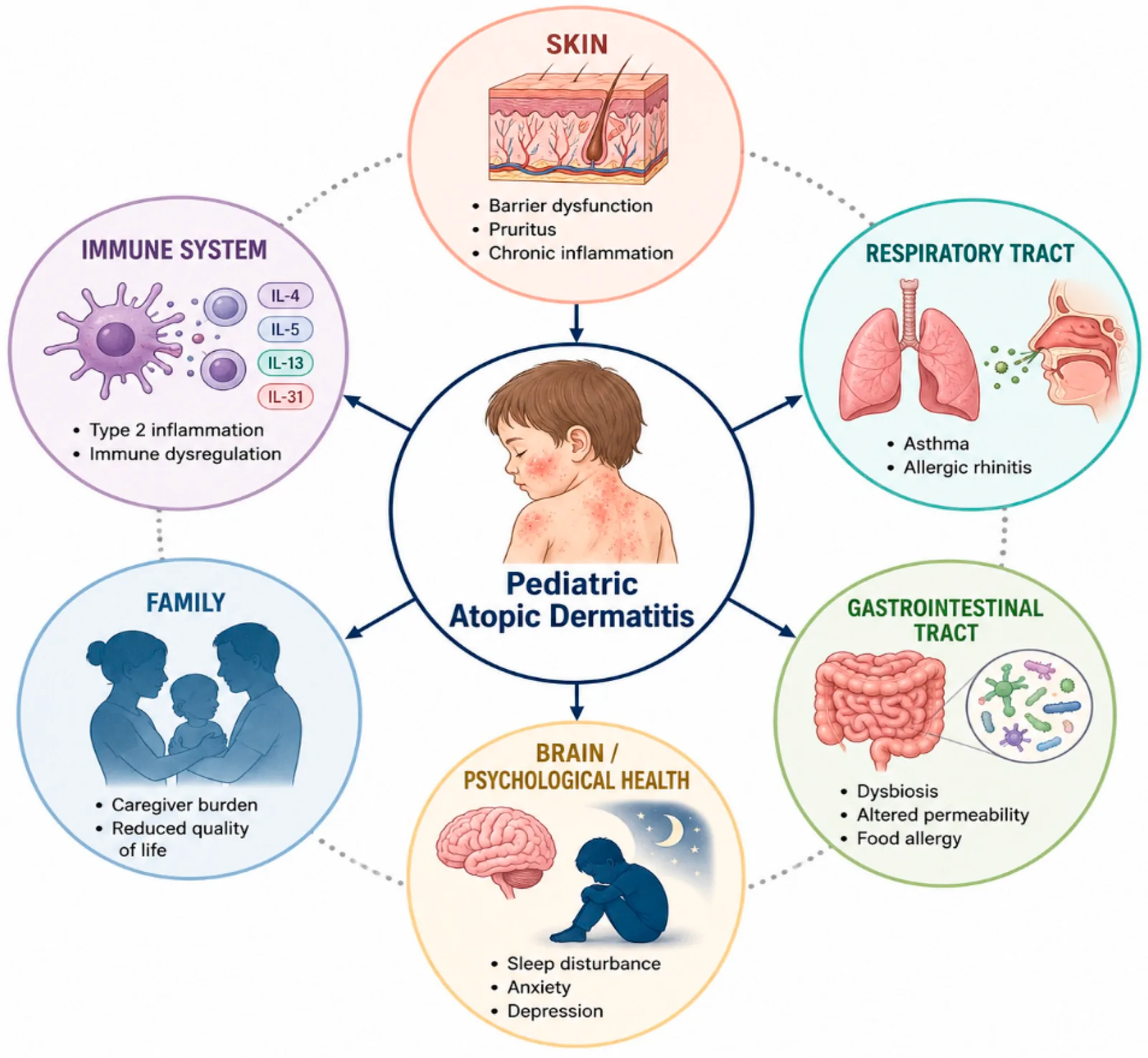
Pediatric atopic dermatitis as a systemic disorder: principal clinical manifestations and comorbidities. The diagram illustrates the multisystem nature of pediatric atopic dermatitis, highlighting immune-mediated comorbidities (allergic respiratory disease, food allergy) alongside gastrointestinal disturbances and the consequences of chronic disease burden (sleep impairment, psychological morbidity, caregiver burden). Together, these manifestations support multidisciplinary management; they do not by themselves establish generalized immune dysregulation. Created using Inkscape (v0.92 and v1.0; Inkscape Project, https://inkscape.org, accessed on 10 September 2026).

**Figure 4:**
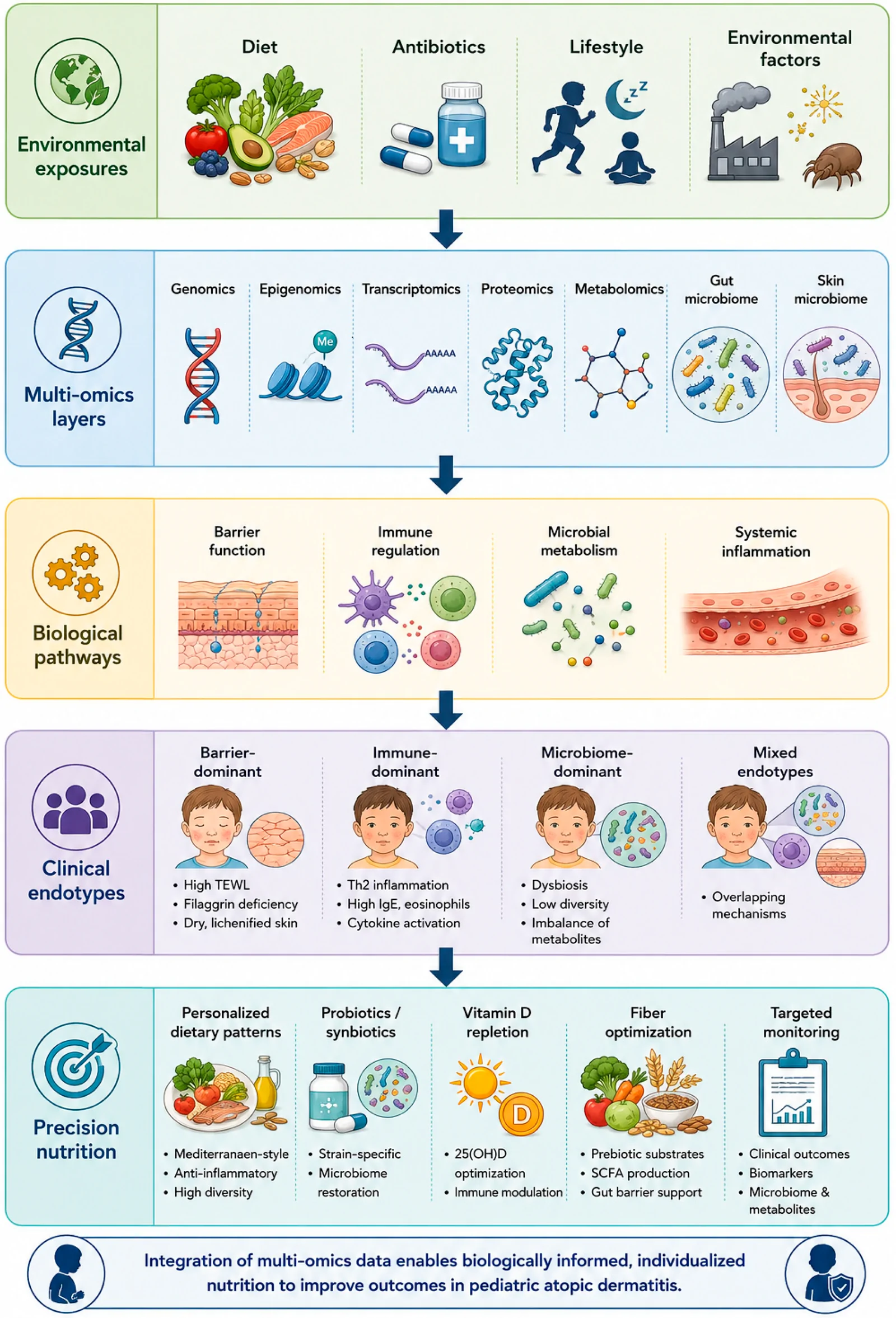
Multi-omics framework supporting precision nutrition in pediatric atopic dermatitis. Environmental and dietary exposures influence multiple interconnected biological layers, including genomics, transcriptomics, proteomics, metabolomics, and the gut and skin microbiomes. Integration of these datasets is proposed as a means of identifying disease endotypes characterized by distinct patterns of barrier dysfunction, immune activation, microbial metabolism, and systemic inflammation. This systems biology approach is a conceptual framework for future precision nutrition research; omics-defined subgroups have not yet been shown to predict responses to dietary interventions in children. Created using Inkscape (v0.92 and v1.0; Inkscape Project, https://inkscape.org, accessed on 10 September 2026).

**Figure 5:**
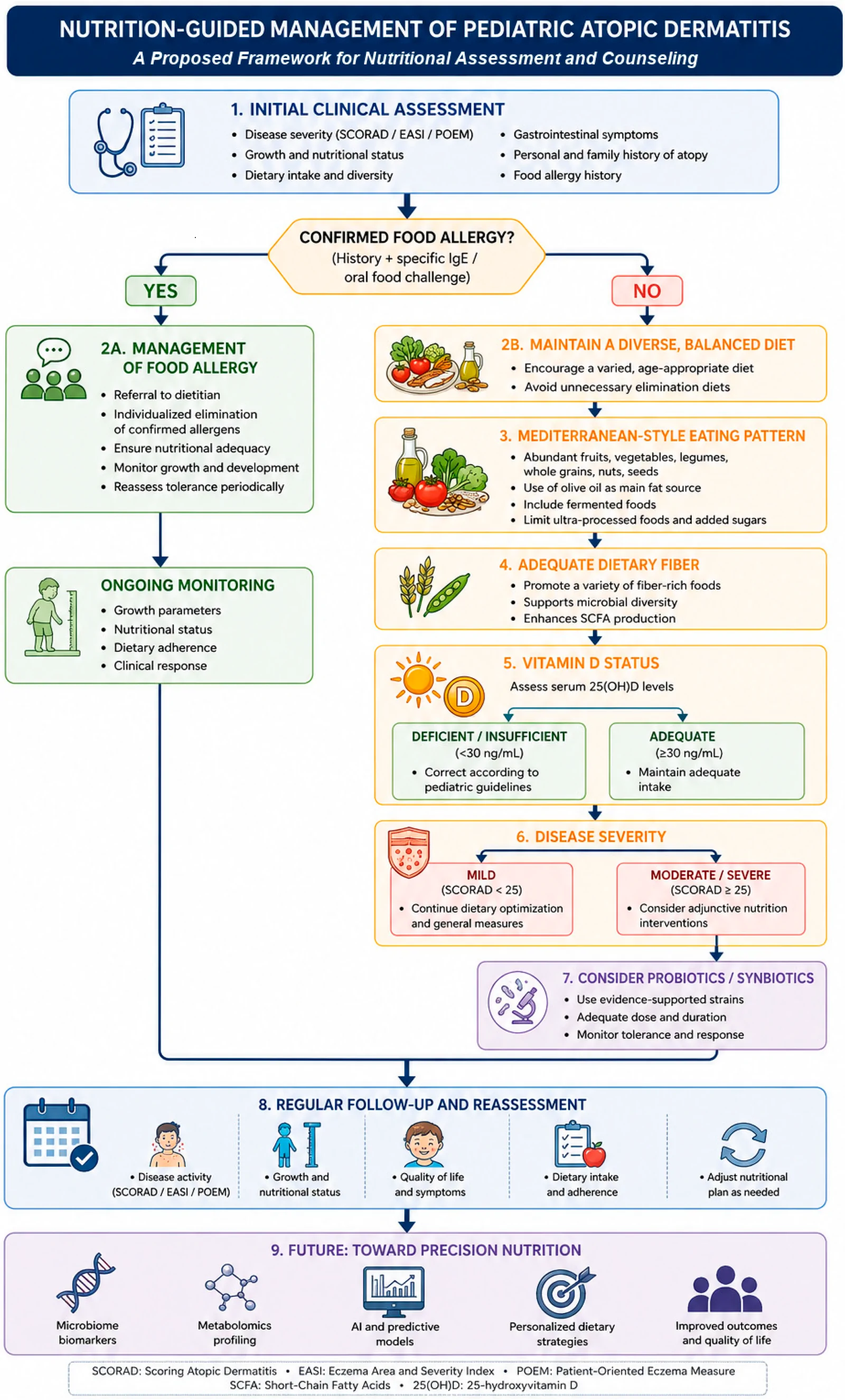
Proposed framework for nutritional assessment and counseling in pediatric atopic dermatitis (author-proposed; not prospectively validated). Created using Inkscape (v0.92 and v1.0; Inkscape Project, https://inkscape.org, accessed on 10 September 2026).

**Table 1:** Clinical phenotypes, sensitization-based subgroups, molecular endotypes, candidate microbial profiles, and potential implications for nutrition-guided management in pediatric atopic dermatitis.

Category and Label	Characteristics	Predominant Biological Features	Potential Nutritional Implications	Key References
Clinical phenotype: early-onset persistent AD	Onset <2 years, severe disease	Strong Th2 activation, FLG defects, dysbiosis	Hypothetical target for early microbiome-directed interventions (untested)	[13,15,18]
Clinical phenotype: transient childhood AD	Mild disease, spontaneous remission	Preserved barrier, limited immune activation	General healthy dietary recommendations	[13,18]
Sensitization-based subgroup: extrinsic (IgE-associated) AD	High IgE, food allergy	Type 2 inflammation	Careful nutritional assessment, avoid unnecessary elimination diets	[50,51,52]
Sensitization-based subgroup: intrinsic (non-IgE-associated) AD	Normal IgE	Mixed immune pathways	Personalized evaluation	[14,52]
Molecular endotype: barrier-dominant (FLG-associated)	FLG mutations, xerosis	Increased TEWL, impaired epidermal integrity	Nutrition supporting barrier function (vitamin D, omega-3 fatty acids, adequate protein intake)	[13,53,54]
Candidate microbial profile (not a validated endotype)	Gut and/or skin dysbiosis	Reduced SCFAs, *S. aureus* dominance	Hypothesized benefit from probiotics, prebiotics, synbiotics, or high-fiber diet; no responder-defining biomarker validated	[30,31,32,55]

AD, atopic dermatitis; FLG, filaggrin; IgE, immunoglobulin E; SCFA, short-chain fatty acid; TEWL, transepidermal water loss. Key references are illustrative (2–4 per row) rather than exhaustive. Evidence descriptors in Table 1, Table 2, Table 3, Table 4 and Table 5 reflect the authors’ narrative appraisal and are not formal GRADE certainty ratings.

**Table 2:** Summary of reported nutrition–microbiome associations within the gut–skin axis in pediatric atopic dermatitis.

Nutritional Factor	Primary Microbiome Target	Proposed Mechanism Within the Gut–Skin Axis	Associated Clinical Outcome	Predominant Type of Supporting Evidence (Narrative Appraisal)	Future Research Priority	Key References
Dietary fiber/Mediterranean dietary pattern	↑ Butyrate-producing bacteria (Faecalibacterium, Roseburia, Coprococcus)	Increased SCFA production promotes Treg differentiation, IL-10 secretion, tight junction integrity, and keratinocyte filaggrin and ceramide synthesis	Association with lower disease severity and lower risk of allergic sensitization in observational studies	Observational and mechanistic; limited randomized data	High	[38,45,56,57]
Vitamin D	↑ Microbial diversity; vitamin D receptor (VDR) signaling	Enhanced antimicrobial peptide production, improved Treg/Th17 balance, and restoration of epidermal barrier gene expression	Reduction in disease severity in randomized trials of children and adults; effect small to moderate and heterogeneous	Randomized and meta-analysed; baseline vitamin D status inconsistently reported	High	[23,58]
Probiotics/Synbiotics (selected strains)	Restoration of beneficial microbial taxa; increased SCFA and indole production	Promotion of Treg differentiation, strengthening of epithelial barrier function, reduction in pathobionts and type 2 inflammatory responses	Modest improvement in disease severity; greater benefit reported with synbiotics and longer treatment duration	Randomized trials and meta-analyses; modest effect, substantial heterogeneity	High	[23,59,60,61]
Breastfeeding/Human milk oligosaccharides (HMOs)	↑ Bifidobacterium infantis and cross-feeding butyrate-producing bacteria	Acetate and lactate production, reduced intestinal pH, promotion of immune tolerance, direct immunomodulatory effects of HMOs	Lower risk of developing atopic dermatitis and allergic sensitization; supportive evidence from HMO-enriched infant formula studies	Consistent observational data; interventional data limited to HMO-supplemented formula	Medium	[39,40,62,63]
Omega-3 fatty acids (EPA/DHA)	Modest increase in microbial diversity; synergistic effects with dietary fiber	Altered membrane lipid composition, specialized pro-resolving mediator synthesis, modulation of eicosanoid pathways	Inconsistent intervention results; observational studies suggest a preventive role	Inconsistent randomized data; some observational support	Medium	[38,63]
Polyphenol-rich and fermented foods	Increased abundance of beneficial taxa and microbial diversity; enhanced SCFA production	Prebiotic-like effects, antioxidant activity, reinforcement of epithelial barrier function, modulation of inflammatory pathways	Supportive observational evidence with limited intervention studies	Mainly observational and mechanistic	Medium	[57,64,65]

EPA, eicosapentaenoic acid; DHA, docosahexaenoic acid; HMO, human milk oligosaccharide; IL, interleukin; SCFA, short-chain fatty acid; Treg, regulatory T cell; VDR, vitamin D receptor. Evidence descriptors reflect the authors’ narrative appraisal and are not formal GRADE certainty ratings; key references are illustrative rather than exhaustive; ↑, indicates an increased relative abundance.

**Table 3:** Practical nutritional considerations according to clinical scenarios in pediatric atopic dermatitis.

Clinical Scenario	Recommended Nutritional Approach	Microbiome Rationale	Level of Evidence (Narrative Appraisal)	Key References
Mild AD without food allergy	Mediterranean-style diet, adequate fiber, diverse complementary feeding	Supports microbial diversity and SCFA production	Low-Moderate (observational)	[38,66,67]
Moderate AD	Assess vitamin D status and correct documented deficiency; a multi-strain probiotic or synbiotic may be discussed as an optional adjunct	Immune modulation and barrier support	Moderate for correction of vitamin D deficiency; Low-Moderate for probiotics/synbiotics	[23,58,60]
Confirmed IgE-mediated food allergy	Individualized elimination diet under dietitian supervision	Prevent nutritional deficiencies while preserving microbiome diversity	High	[68,69,70]
Recent antibiotic exposure	Encourage prebiotic-rich foods and fermented products	Facilitate microbial recovery	Low (no trials in pediatric atopic dermatitis)	[71,72]
Vitamin D deficiency	Correct deficiency according to pediatric guidelines	Barrier and immune support	Moderate	[23,58]

AD, atopic dermatitis; SCFA, short-chain fatty acid. Evidence descriptors reflect the authors’ narrative appraisal and are not formal GRADE certainty ratings; key references are illustrative rather than exhaustive.

**Table 4:** Candidate biomarkers supporting precision nutrition in pediatric atopic dermatitis.

Biomarker Category	Representative Biomarkers	Principal Clinical Utility	Strength of Current Evidence	Current Clinical Applicability	BEST Category (FDA-NIH) [73]	Key References
Clinical	SCORAD, EASI, POEM	Disease severity and treatment monitoring	High	Routine	Monitoring; response	[52,74]
Immune	Total IgE, eosinophils, TARC, IL-13, IL-31	Endotyping and inflammatory profiling	Moderate	Emerging	Diagnostic; monitoring; candidate prognostic	[52,75,76]
Barrier	TEWL, filaggrin, ceramides	Assessment of epidermal barrier integrity	Moderate	Emerging	Diagnostic; monitoring; candidate susceptibility (infant tape strips)	[53,77,78]
Gut microbiome	Shannon index, α-diversity, *Faecalibacterium*, *Roseburia*	Risk stratification and patient phenotyping	Moderate	Research	Candidate susceptibility/risk and prognostic; not predictive	[30,31,79]
Microbial metabolites	SCFAs, indoles, secondary bile acids	Functional microbiome assessment; proposed (unvalidated) prediction of nutritional response	Moderate	Research	Candidate susceptibility and exposure; not validated as predictive	[31,79,80]
Intestinal permeability	Zonulin, LBP, I-FABP, Reg3A	Identification of gut barrier dysfunction	Low–Moderate	Research	Exploratory; category not yet established	[81,82]
Nutritional	25(OH)D, omega-3 index, dietary fiber intake	Nutritional assessment and individualized supplementation	High (vitamin D); Moderate (others)	Routine/Emerging	Exposure (dietary intake); diagnostic of deficiency (25(OH)D); not validated as predictive of response to a nutritional intervention	[23,38,58]

25(OH)D, 25-hydroxyvitamin D; BEST, Biomarkers, EndpointS, and other Tools; EASI, Eczema Area and Severity Index; I-FABP, intestinal fatty acid-binding protein; IL, interleukin; LBP, lipopolysaccharide-binding protein; POEM, Patient-Oriented Eczema Measure; Reg3A, regenerating islet-derived protein 3 alpha; SCFA, short-chain fatty acid; SCORAD, SCORing Atopic Dermatitis; TARC, thymus and activation-regulated chemokine; TEWL, transepidermal water loss. ‘Predictive’ is used in the BEST sense (prediction of response to a specific intervention); no marker listed has been validated as predictive of response to a nutritional intervention in children. Evidence descriptors reflect the authors’ narrative appraisal and are not formal GRADE certainty ratings; key references are illustrative rather than exhaustive.

**Table 5:** Nutrition-based interventions targeting the gut–skin axis in pediatric atopic dermatitis: mechanisms, evidence, and clinical recommendations.

Intervention	Proposed Mechanism Within the Gut–Skin Axis	Current Evidence	Clinical Recommendation	Key Research Priorities	Key References
Breastfeeding and HMOs	Promotion of *Bifidobacterium*, SCFA production, immune tolerance	Consistent observational evidence; limited trial data for HMO-supplemented formulas	Encourage exclusive breastfeeding when possible; when it is not feasible, select formula according to pediatric nutrition guidance, since HMO supplementation has not been shown to prevent or treat atopic dermatitis	Optimal HMO composition and long-term effects on AD prevention	[39,40,63,83]
Probiotics/Synbiotics	Modulation of microbial composition, SCFA production, Treg induction, barrier support	Randomized evidence; pooled SCORAD reduction small, below the minimal clinically important difference, with substantial heterogeneity	Not recommended as treatment by current guidelines; may be discussed as an optional adjunct in moderate-to-severe disease, with realistic expectations and a predefined trial period	Identification of optimal strains, doses, treatment duration, and responder profiles	[23,59,60,61]
Vitamin D repletion	Barrier integrity, immune regulation, potential microbiome modulation	Meta-analysis of 11 randomized trials shows reduced severity; trials small and heterogeneous, with baseline vitamin D status inconsistently reported	Assess vitamin D status and correct deficiency according to pediatric guidelines	Optimal dosing strategies and interaction with microbiome composition	[23,58]
High-fiber/Mediterranean dietary pattern	Increased microbial diversity, SCFA production, anti-inflammatory metabolites	Observational evidence only; no randomized dietary-pattern trials in pediatric atopic dermatitis	Promote a plant-rich, fiber-rich dietary pattern with fermented foods; reduce ultra-processed foods	Well-designed randomized dietary intervention trials incorporating microbiome and metabolomic outcomes	[38,45,57,67]

AD, atopic dermatitis; HMO, human milk oligosaccharide; SCFA, short-chain fatty acid; Treg, regulatory T cell. Evidence descriptors reflect the authors’ narrative appraisal and are not formal GRADE certainty ratings; key references are illustrative rather than exhaustive.

## Data Availability

No new data were created or analyzed in this study. Data sharing is not applicable to this article.

## Supplementary Materials

The following supporting information can be downloaded at: https://www.mdpi.com/article/10.3390/nu18193138/s1, Table S1: Complete search strings, fields, and dates used for the literature search.

## Abbreviations

The following abbreviations are used in this manuscript:

25(OH)D	25-Hydroxyvitamin D
AD	Atopic dermatitis
AhR	Aryl hydrocarbon receptor
AI	Artificial intelligence
BEST	Biomarkers, EndpointS, and other Tools
DHA	Docosahexaenoic acid
EAACI	European Academy of Allergy and Clinical Immunology
EASI	Eczema Area and Severity Index
EPA	Eicosapentaenoic acid
FLG	Filaggrin
FXR	Farnesoid X receptor
HMO	Human milk oligosaccharide
I-FABP	Intestinal fatty acid-binding protein
3-IAld	Indole-3-carboxaldehyde
IgE	Immunoglobulin E
IL	Interleukin
ILA	Indole-3-lactic acid
ISAPP	International Scientific Association for Probiotics and Prebiotics
JAK	Janus kinase
LBP	Lipopolysaccharide-binding protein
MeSH	Medical Subject Headings
ML	Machine learning
POEM	Patient-Oriented Eczema Measure
Reg3A	Regenerating islet-derived protein 3 alpha
SCFA	Short-chain fatty acid
SCORAD	SCORing Atopic Dermatitis
TARC/CCL17	Thymus and activation-regulated chemokine/C-C motif chemokine ligand 17
TEWL	Transepidermal water loss
TGF-β	Transforming growth factor beta
TGR5	Takeda G protein-coupled receptor 5
Th	T helper
Treg	Regulatory T cell
TSLP	Thymic stromal lymphopoietin
VDR	Vitamin D receptor
