# Supplementary material for: The Gut–Skin Axis in Pediatric Atopic Dermatitis: Nutritional Modulation, Microbiome Interactions, and Emerging Precision Approaches
Source: Nutrients. 2026 Sep 23;18(19):3138. doi: 10.3390/nu18193138 (PMC13667208; doi:10.3390/nu18193138)
Supplement: Supplementary file 1 [file nutrients-18-03138-s001.zip › nutrients-4550414-supplementary.pdf]

Supplementary Table S1. Complete search strings, fields, and dates used for the literature search

Manuscript: The Gut-Skin Axis in Pediatric Atopic Dermatitis: Nutritional Modulation, Microbiome Interactions, and Emerging Precision Approaches (Nutrients, manuscript ID nutrients-4550414). Date range: 1 January 2018 to 31 July 2026; last search update 31 July 2026. Language: English. Earlier landmark publications were retained through manual screening of reference lists.

Database	Fields searched	Search string	Date of last search
PubMed/MEDLINE	MeSH terms; title/abstract [tiab]; publication date [dp]; language [la]	("dermatitis, atopic"[MeSH] OR "atopic dermatitis"[tiab] OR eczema[tiab]) AND (child*[tiab] OR pediatric[tiab] OR paediatric[tiab] OR infant*[tiab] OR "Child"[MeSH]) AND ("gastrointestinal microbiome"[MeSH] OR "gut microbio*"[tiab] OR "gut-skin axis"[tiab] OR "skin microbio*"[tiab] OR dysbiosis[tiab] OR probiotic*[tiab] OR prebiotic*[tiab] OR synbiotic*[tiab] OR postbiotic*[tiab] OR "short-chain fatty acid*"[tiab] OR indole*[tiab] OR "bile acid*"[tiab] OR metabolom*[tiab] OR "multi-omics"[tiab] OR "machine learning"[tiab] OR "artificial intelligence"[tiab] OR biomarker*[tiab]) AND (diet*[tiab] OR nutrition*[tiab] OR breastfeed*[tiab] OR "complementary feeding"[tiab] OR "Mediterranean diet"[tiab] OR "food allergy"[tiab] OR "elimination diet"[tiab] OR "vitamin D"[tiab] OR "omega-3"[tiab] OR "precision nutrition"[tiab] OR "personalized nutrition"[tiab]) AND ("2018/01/01"[dp] : "2026/07/31"[dp]) AND english[la]	31 July 2026
Scopus	TITLE-ABS-KEY; PUBYEAR; LANGUAGE	TITLE-ABS-KEY(("atopic dermatitis" OR eczema) AND (child* OR pediatric OR paediatric OR infant*) AND ("gut microbio*" OR "gut-skin axis" OR "skin microbio*" OR dysbiosis OR probiotic* OR prebiotic* OR synbiotic* OR postbiotic* OR "short-chain fatty acid*" OR indole* OR "bile acid*" OR metabolom* OR "multi-omics" OR "machine learning" OR "artificial intelligence" OR biomarker*) AND (diet* OR nutrition* OR breastfeed* OR	31 July 2026

		"complementary feeding" OR "Mediterranean diet" OR "food allergy" OR "elimination diet" OR "vitamin D" OR "omega-3" OR "precision nutrition" OR "personalized nutrition")) AND PUBYEAR > 2017 AND PUBYEAR < 2027 AND LANGUAGE(english)	
Web of Science	Topic (TS: title, abstract, author keywords, Keywords Plus); PY; LA	TS=(("atopic dermatitis" OR eczema) AND (child* OR pediatric OR paediatric OR infant*) AND ("gut microbio*" OR "gut-skin axis" OR "skin microbio*" OR dysbiosis OR probiotic* OR prebiotic* OR synbiotic* OR postbiotic* OR "short-chain fatty acid*" OR indole* OR "bile acid*" OR metabolom* OR "multi-omics" OR "machine learning" OR "artificial intelligence" OR biomarker*) AND (diet* OR nutrition* OR breastfeed* OR "complementary feeding" OR "Mediterranean diet" OR "food allergy" OR "elimination diet" OR "vitamin D" OR "omega-3" OR "precision nutrition" OR "personalized nutrition")) AND PY=(2018-2026) AND LA=(English); Web of Science Core Collection (SCI-EXPANDED, SSCI, ESCI)	31 July 2026

Boolean operators: AND between concept blocks, OR within blocks. Truncation: asterisk (*) for word stems. Reference lists of included guidelines, systematic reviews, and meta-analyses were screened manually. Records were screened independently by two authors (R.-G.M. and C.M.), with disagreements resolved by discussion with a third author (O.A.C.). As this is a narrative review, no PRISMA flow diagram was produced and record counts at each stage were not formally tracked.
